# Development of a 4-aminopyrazolo[3,4-*d*]pyrimidine-based dual IGF1R/Src inhibitor as a novel anticancer agent with minimal toxicity

**DOI:** 10.1186/s12943-018-0802-4

**Published:** 2018-02-19

**Authors:** Ho Jin Lee, Phuong Chi Pham, Seung Yeob Hyun, Byungyeob Baek, Byungjin Kim, Yunha Kim, Hye-Young Min, Jeeyeon Lee, Ho-Young Lee

**Affiliations:** 1 Creative Research Initiative Center for Concurrent Control of Emphysema and Lung Cancer, College of Pharmacy, Seoul National University, Seoul, 08826 Republic of Korea; 2 College of Pharmacy and Research Institute of Pharmaceutical Sciences, Seoul National University, Seoul, 08826 Republic of Korea; 3 Department of Molecular Medicine and Biopharmaceutical Science, Graduate School of Convergence Science and Technology, and College of Pharmacy, Seoul National University, Seoul, 08826 Republic of Korea

## Abstract

**Background:**

Both the type I insulin-like growth factor receptor (IGF1R) and Src pathways are associated with the development and progression of numerous types of human cancer, and Src activation confers resistance to anti-IGF1R therapies. Hence, targeting both IGF1R and Src concurrently is one of the main challenges in combating resistance to the currently available anti-IGF1R-based anticancer therapies. However, the enhanced toxicity from this combinatorial treatment could be one of the main hurdles for this strategy, suggesting the necessity of developing a novel strategy for co-targeting IGF1R and Src to meet an urgent clinical need.

**Methods:**

We synthesized a series of 4-aminopyrazolo[3,4-*d*]pyrimidine-based dual IGF1R/Src inhibitors, selected LL28 as an active compound and evaluated its potential antitumor effects in vitro and in vivo using the MTT assay, colony formation assays, flow cytometric analysis, a tumor xenograft model, and the *Kras*^*G12D/+*^-driven spontaneous lung tumorigenesis model.

**Results:**

LL28 markedly suppressed the activation of IGF1R and Src and significantly inhibited the viability of several NSCLC cell lines in vitro by inducing apoptosis. Administration of mice with LL28 significantly suppressed the growth of H1299 NSCLC xenograft tumors without overt toxicity and substantially reduced the multiplicity, volume, and load of lung tumors in the *Kras*^*G12D/+*^-driven lung tumorigenesis model.

**Conclusions:**

The present results suggest the potential of LL28 as a novel anticancer drug candidate targeting both IGF1R and Src, providing a new avenue to efficient anticancer therapies. Further investigation is warranted in advanced preclinical and clinical settings.

**Electronic supplementary material:**

The online version of this article (10.1186/s12943-018-0802-4) contains supplementary material, which is available to authorized users.

## Background

Cancer is one of the main cause of human deaths globally. Among the various kinds of cancer, lung cancer is one of the worst cancer types in terms of incidence and mortality [1]. Despite extensive efforts to cure lung cancer, the 5-year survival rate of lung cancer is still less than 20%, mostly due to diagnosis occurring at a late stage and resistance to anticancer therapies. Currently used anticancer treatments for lung cancer, including conventional chemotherapy and molecular targeted therapy, inevitably elicits the development of drug resistance through multiple mechanisms, which is a major obstacle to effe ctive anticancer treatment. Therefore, establishing a therapeutic strategy to hamper the development of drug resistance would be essential for effective anticancer therapy.

The type I insulin-like growth factor receptor (IGF1R) signaling plays an important role in cell transformation, proliferation, survival, metastasis [2, 3], and resistance to various anticancer therapies [4]. Insulin-like growth factor 1 (IGF1)-IGF1R signaling induces the expression of vascular endothelial growth factor (VEGF) in hypoxia-inducible factor-1α (HIF1α)-dependent and HIF1α-independent manners [4, 5] and interacts with other receptor tyrosine kinases, further enhancing signaling pathways promoting cell proliferation and survival [4]. Activation of IGF1R has been found in several types of human cancer, including lung cancer [4]. Hyperactivation of IGF1R signaling has been associated with resistance to radiotherapy and chemotherapies, including paclitaxel and cisplatin [6–9]. Heterodimerization between IGF1R and epidermal growth factor receptor (EGFR) mediates the activation of bypass signaling, leading to resistance to gefitinib and erlotinib in lung cancer [10, 11]. Reprogramming IGF1R has also been implicated in adaptive resistance to various molecularly targeted therapies, such as trastuzumab (an anti-HER2/neu receptor monoclonal antibody), lapatinib [an anti-HER2 tyrosine kinase inhibitor (TKI)], SB-590885 (a BRAF inhibitor), and crizotinib [an anaplastic lymphoma kinase (ALK) TKI] [12–15]. These findings have indicated IGF1R signaling as an attractive target for anticancer therapy. However, the efficacy of IGF1R-targeted therapies, which mainly include monoclonal antibodies (mAbs) and TKIs, have been marginal in a variety of clinical trials, and many of these clinical trials have been halted due to poor clinical outcomes [16]. Previous studies have demonstrated the involvement of Src in resistance to IGF1R inhibitors [17–19]. Indeed, combined treatment with IGF1R and Src inhibitors has shown significantly improved anticancer activities compared with single drug treatments [17–20]. However, considering the potentially increased toxicities resulting from the combined use of the two inhibitors, developing dual kinase inhibitors concurrently targeting IGF1R and Src would be more beneficial. In this regard, we and others recently reported oxadiazinones and oxoacetohydrazides as dual IGF1R/Src inhibitor scaffolds [21, 22]. In fact, several reports have proposed the potential of multifunctional compounds to hit more than one target due to the limitation of the traditional “one molecule, one target” paradigm in drug development as well as in clinical trials. The success of the pharmacophore combination approach is highly dependent on the attachment position, physical properties, and length of the spacer.

Here, we report the discovery of LL28, a 4-aminopyrazolo[3,4-*d*]pyrimidines-based dual IGF1R/Src inhibitor that exhibits effective anticancer activity in vitro and in vivo. LL28 effectively inhibits both IGF1R- and Src-dependent signaling pathways and has promising therapeutic potential in vitro against a panel of non-small cell lung cancer (NSCLC) cell lines and in vivo in tumor xenograft and mutant *Kras*-driven lung tumorigenesis models with marginal toxicity.

## Results

### Synthesis of LL28

**Fig. 1:**
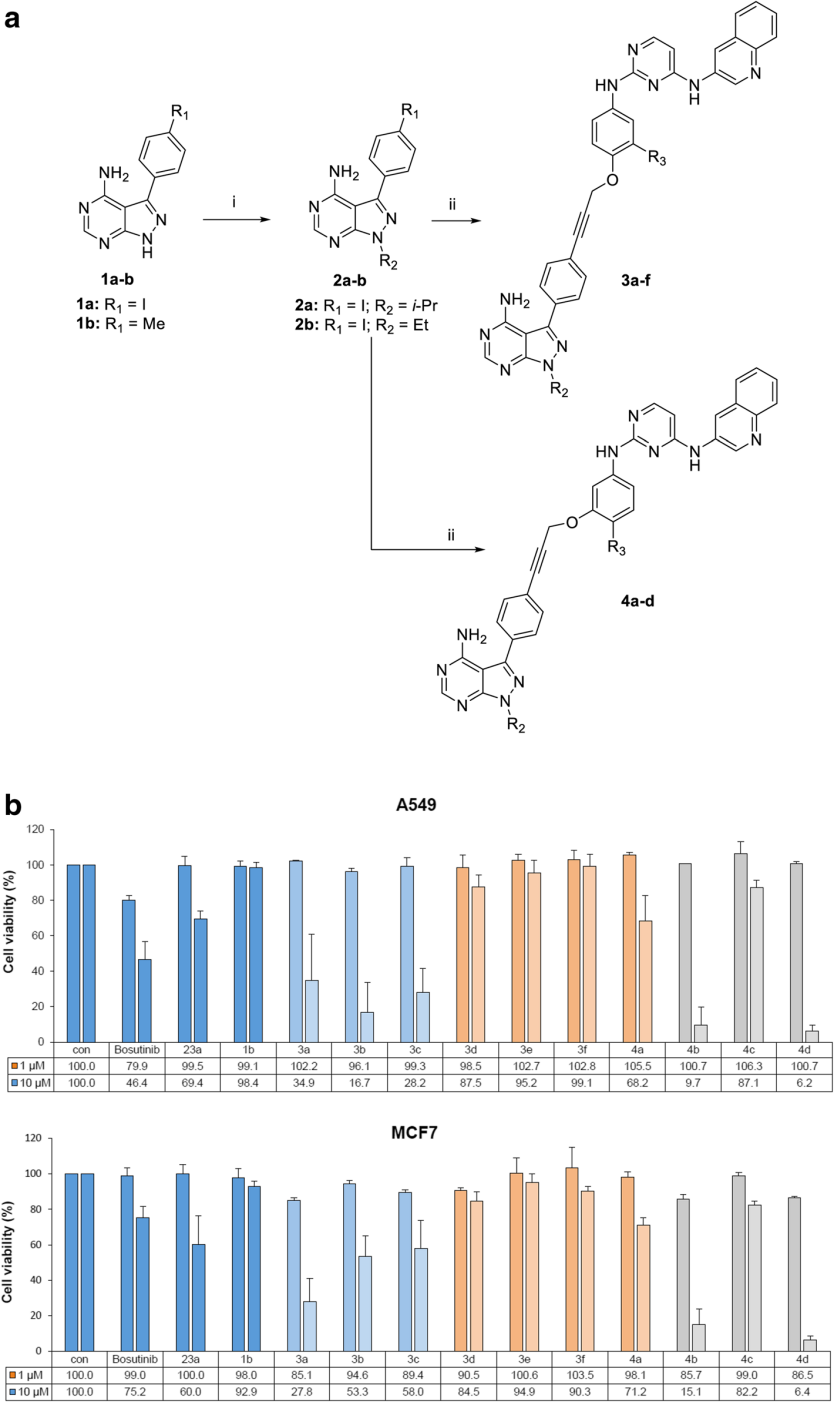
Synthesis and screening of compounds 3a-f and 4a-d for anticancer activity. **a** Reagents and conditions: i) R_2_I, K_2_CO_3_, Dimethylformamide, 60 °C, 3 h, 51-56%; ii) 24a-e, Pd(PPh_3_)_2_Cl_2_, CuI, Triethylamine, Dimethylformamide, rt., 3 h, 40-72%. **b** Inhibitory effects of compounds **3a-f** and **4a-d** on the viability of A549 and MCF7 cells were evaluated by the MTT assay. The bars represent the means ± SD

The 4-aminopyrazolo[3,4-*d*]pyrimidines, a well-characterized class of compounds for tyrosine kinase inhibition, were used as a Src inhibitor module that blocks the adenosine binding site [23–26]. A 2,4-bis-arylamino-1,3-pyrimidines module was used for anti-IGF1R activity [27]. Recently, an alkyne motif has been applied successfully in several TKIs, including ponatinib [28, 29]. Ponatinib possesses an alkyne linker between the imidazo[1,2-*b*]pyridazine and diarylamide (a 2,3-diarylethyne motif) and a slim alkyne moiety was found to be crucial to activity by avoiding steric hindrance [30]. Using the pharmacophore combination strategy, we synthesized a series of new compounds **3a-f** and **4a-d** bearing an alkyne linker using the palladium-catalyzed Sonogashira coupling reaction as a key step (Fig. 1a and Table 1). Details of the synthesis and characterization of compounds **3a-f**, **4a-d**, and their intermediate compounds are depicted in the supplementary information (Additional file 1).

**Table 1:** Structures of compound **3a-f** and **4a-d** and antiproliferative activity against cancer cell lines

Compd	linkage	R_2_	R_3_	% cells growth at 10 μM	
				A549	MCF-7
3a	para	*i*-Pr	H	34	27
3b		*i-Pr*	Cl	16	53
3c		*i-Pr*	F	28	58
3d		Et	H	87	84
3e		Et	Cl	95	94
3f		Et	F	99	90
4a	meta	*i-Pr*	H	68	71
4b		*i-Pr*	OMe	9.7	15
4c		Et	H	87	82
4d		Et	OMe	6.2	6.4
Bosutinib				46	75
1b				98	92
23a				69	60

We next tested the effects of compounds **3a-f** and **4a-d** on the viability of A549 and MCF7 cells and compared their inhibitory activity with that of fragmented Src and IGF1R modules (**1b** and **23a**) as controls. Interestingly, the addition of a methoxy group to the para position of the aminophenol ring significantly increased the overall potency against both cancer cell lines compared with those of unsubstituted compounds (Fig. 1b). Compounds **4b** and **4d** with a p-methoxy group at the R_3_ position exhibited significant cell growth inhibition at a concentration of 10 μM. Compounds featuring different attachments of the alkyne linker at the para-position of aminophenyl ring (**3b** and **3c**) also demonstrated high potency but to a lesser extent than those with the meta-linkage. In the case of para-linked compounds, the replacement of the isopropyl group in compounds **3a-c** with an ethyl group in compounds **3d-f** led to a decrease in inhibitory effects indicating that a bulky group at R_2_ may be required for cytotoxicity. Our results showed that the p-methoxy (alkoxy) groups are critical for inhibition and that proper linkage between two modules can be tuned for better efficacy. Note that the Src and IGF1R modules (**1b** and **23a**) alone did not exhibit potent anticancer activity, demonstrating that dual-targeting compounds are more efficient. Several compounds with antiproliferative activity were selected to determine their half maximal inhibitory concentration (IC_50_) value against two cancer cell lines, A549 and MCF7 (Additional file 2: Table S1), and compound **4b** (**LL28**), which had the lowest IC_50_ value, was selected for further studies.

### LL28 inhibits activation of both IGF1R and Src in human NSCLC cells

**Fig. 2:**
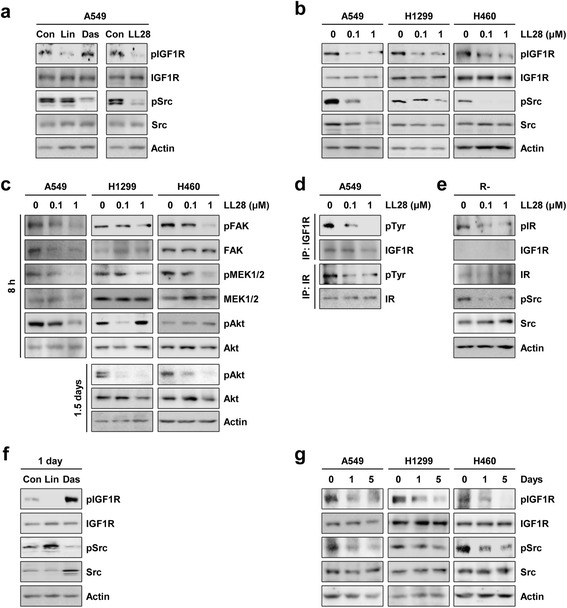
Inhibitory effect of LL28 on the activation of both IGF1R and Src. **a** A549 cells were treated with linsitinib (1 μM), dasatinib (100 nM), or LL28 (1 μM) for 4 h. Before harvesting, cells were stimulated with FBS for 20 min. The expression of total and phosphorylated IGF1R and Src was evaluated by Western blot analysis. (**b** and **c**) A549, H1299, and H460 cells were treated with LL28 (0.1 and 1 μM) for 8 h (**b** and **c**) or 1.5 days (**c**). **b** The expression of total and phosphorylated IGF1R and Src was evaluated by Western blot analysis. **c** The expression of the total and phosphorylated forms of several kinases was evaluated by Western blot analysis. **d** Total cell lysates of A549 cells treated with LL28 for 8 h were immunoprecipitated with anti-IGF1R or anti-IR antibodies. The immunoprecipitants were further subjected to Western blot analysis using anti-pTyr, anti-IGF1R, and anti-IR antibodies. **e** R- cells were treated with LL28 (0.1 and 1 μM) for 8 h. The expression of total and phosphorylated IGF1R and Src was determined by Western blot analysis. **f** A549 cells were treated with linsitinib (1 μM) or dasatinib (100 nM) for 1 day. The expression of total and phosphorylated IGF1R and Src was evaluated by Western blot analysis. **g** A549, H1299, and H460 cells were treated with LL28 (0.1 μM) for 1, 3, and 5 days. The expression of total and phosphorylated IGF1R and Src was evaluated by Western blot analysis. Con: control; Lin: linsitinib; Das: dasatinib

We then investigated the effect of LL28 on the IGF1R and Src activation. We have previously shown effective suppression of IGF1R or Src phosphorylation in NSCLC cell lines by treatment with the small molecule tyrosine kinase inhibitors linsitinib (1 μM) or dasatinib (100 nM), respectively [19]. Hence, A549 cells were treated with linsitinib (1 μM), dasatinib (100 nM), or LL28 (1 μM) diluted in complete medium for 4 h and stimulated with FBS for 20 min before harvesting. Indeed, treatment with linsitinib (1 μM) or dasatinib (100 nM) effectively suppressed IGF1R or Src phosphorylation, respectively, and LL28 (1 μM) induced concurrent suppression of the IGF1R and Src phosphorylation (Fig. 2a). Treatment with LL28 also showed dose-dependent suppression of IGF1R, Src, and their downstream mediators, including FAK, MEK, and Akt, in A549, H1299, and H460 NSCLC cells (Fig. 2b and c). Many IGFIR targeting agents also target the insulin receptor (IR). Therefore, we assessed whether LL28 could also inhibit IR signaling and function. To this end, lysates from A549 cells treated with vehicle or LL28 for 8 h were immunoprecipitated with anti-IGF1R or anti-IR antibodies, and then immunoblot analysis was performed using antibodies against phosphorylated tyrosine (pTyr). The phosphorylation of both IGF1R and IR was markedly inhibited by treatment with LL28 (Fig. 2d). We further confirmed LL28-induced suppression of IR phosphorylation in mouse embryonic fibroblasts (MEF) obtained from *Igf1r* knockout mice (R- cells, expressing only IR) [31] (Fig. 2e). These data suggest that, like other IGF1R TKIs, LL28 also blocks both IGF1R and IR.

We next assessed the communication between the IGF1R and Src signaling pathways in NSCLC cell lines after treatment with linsitinib (1 μM), dasatinib (100 nM), or LL28 (1 μM) for 1 day. As demonstrated in the previous report [19], inhibition of IGF1R by treatment with linsitinib resulted in the activation of Src, and treatment with a Src-family kinase (SFK) inhibitor dasatinib also caused upregulation of IGF1R activation (Fig. 2f). Therefore, it was likely that IGF1R and Src are mutually associated and that inhibition of one kinase leads to the activation of the other kinase as a bypass signaling. In contrast, the inhibitory effects of LL28 (1 μM) on IGF1R and Src phosphorylation were maintained up to 5 days in A549, H1299, and H460 NSCLC cells (Fig. 2g).

### LL28 inhibits the viability and colony forming ability of a number of human NSCLC cells by inducing apoptosis

**Fig. 3:**
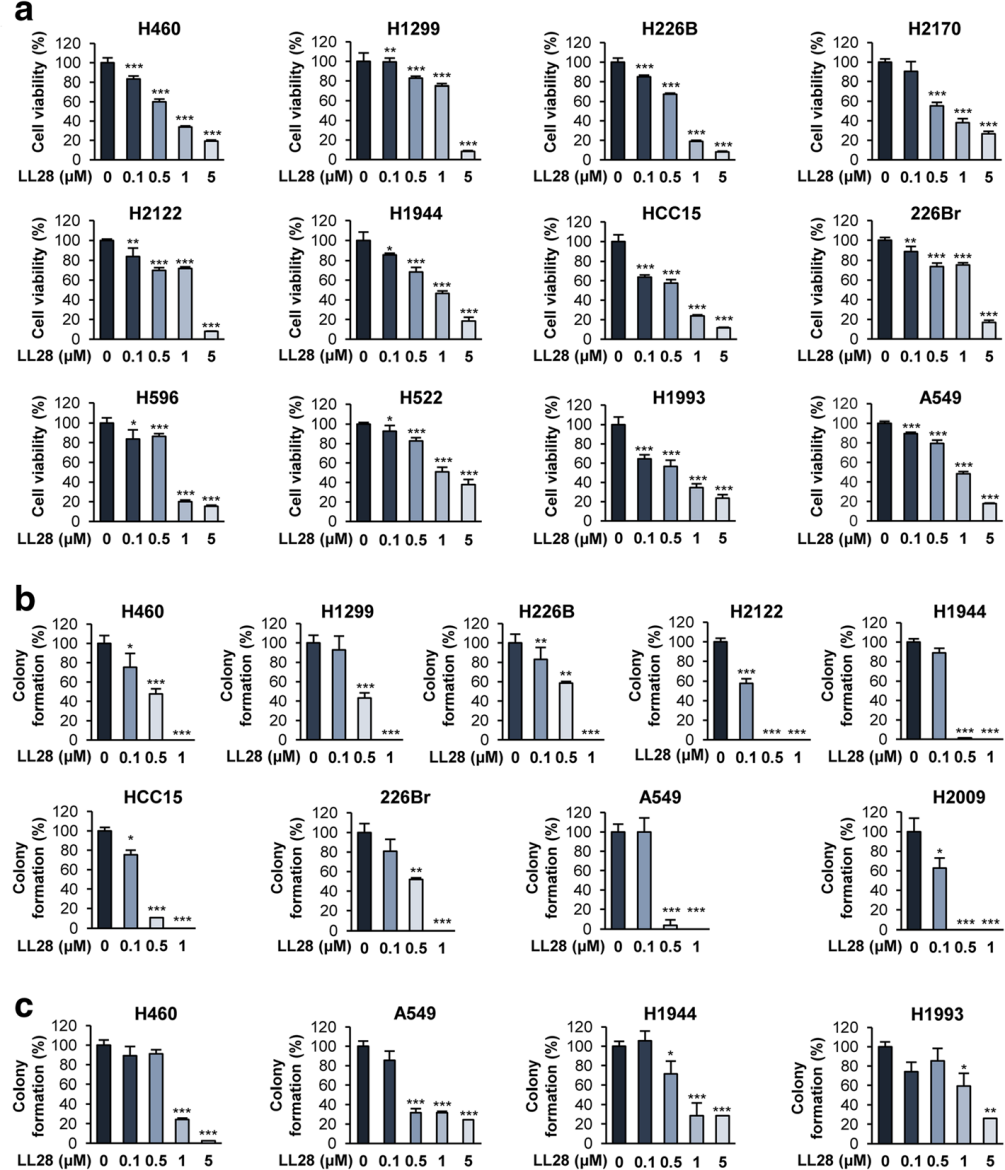
Inhibitory effect of LL28 on the viability and colony forming ability of a panel of lung cancer cells. **a**-**c** The effects of LL28 on the viability (**a**), anchorage-dependent colony formation (**b**), and anchorage-independent colony formation (**c**) of a panel of lung cancer cells were evaluated by the MTT assay (**a**) and colony formation assays under anchorage-dependent (**b**) and anchorage-independent (**c**) culture conditions, as described in the Methods section. The bars represent the means ± SD; *P < 0.05, **P < 0.01, and ***P < 0.001, as determined by a two-sided Student’s *t*-test

We then investigated the efficacy of LL28 in NSCLC cells. We first evaluated the effect of LL28 on the viability and colony forming ability of several NSCLC cell lines in both anchorage-dependent and anchorage-independent culture conditions. LL28 significantly inhibited the viability of NSCLC cells in a dose-dependent manner (Fig. 3a). The IC_50_ value of this compound in each cell line tested was approximately 1 μM on average (Additional file 3: Table S2). Because the genetic backgrounds of these cell lines are varied, this result suggests that LL28 displays a general anticancer effect that is not dependent on a specific genetic alteration. Consistent with these results, LL28 displayed significant and dose-dependent inhibitory effects on colony formation of cells grown in anchorage-dependent and anchorage-independent conditions (Fig. 3b and c). Notably, treatment with LL28 significantly blocked anchorage-dependent colony forming capacity of most of NSCLC cells under adherent conditions, even at a concentration of 0.5 μM (Fig. 3b), and the IC_50_ value of this compound was less than 1 μM in all NSCLC cell lines tested (Additional file 4: Table S3). Thus, considering that clonogenicity under anchorage-dependent conditions is an indicator of cell survival [32], these results indicate that LL28 effectively suppressed NSCLC cell survival.

**Fig. 4:**
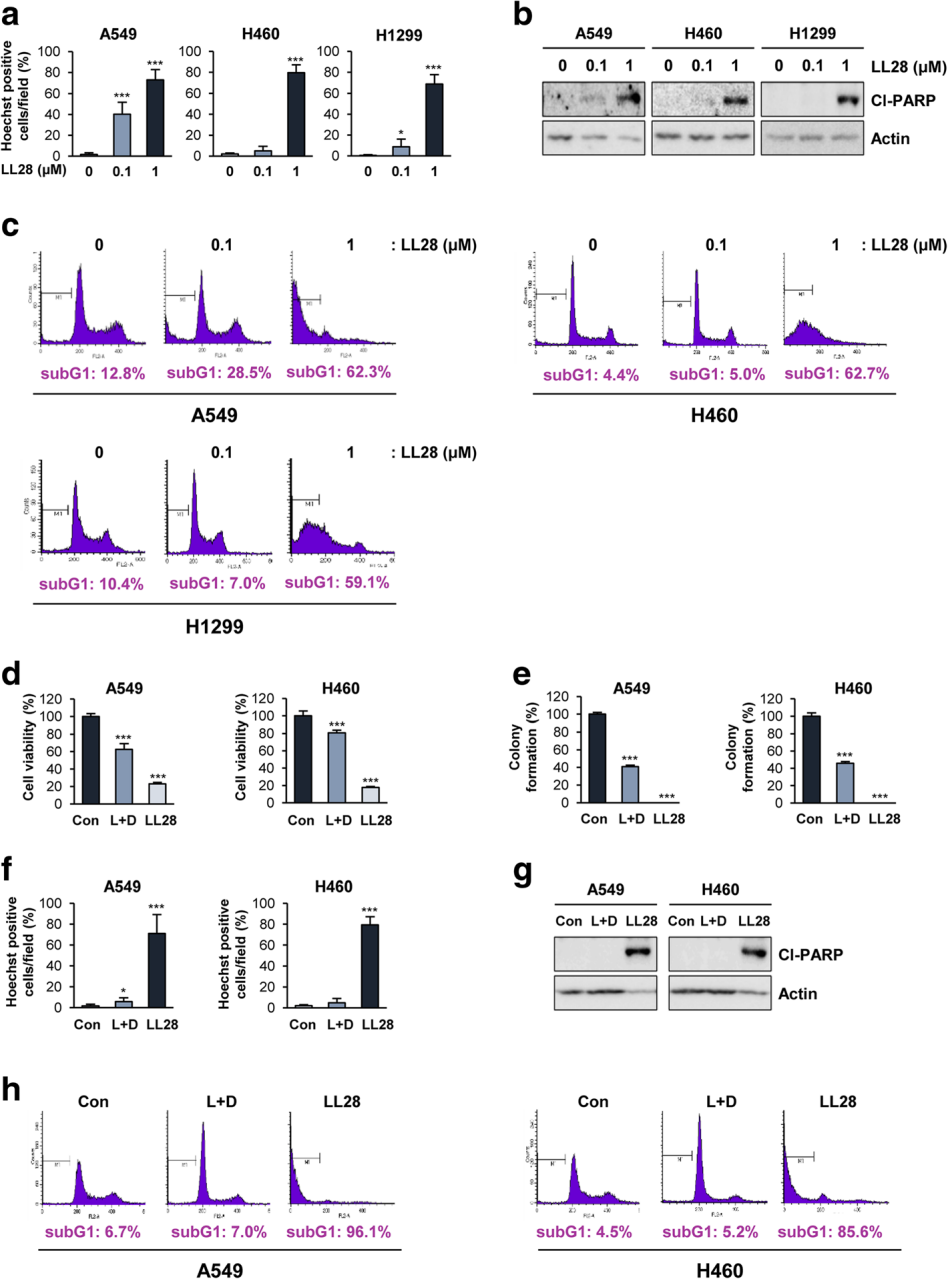
Induction of apoptosis by treatment with LL28. **a**-**c** A549, H460, and H1299 cells were treated with LL28 (0.1 and 1 μM) for 3 days. **a** The chromatin condensation in the nuclei was observed by fluorescence microscopy, photographed, and counted. **b** The expression of cleaved PARP (Cl-PARP) was determined by Western blot analysis. **c** The cell cycle distribution in vehicle- or LL28-treated cells was analyzed by flow cytometry. (D-H) A549 and H460 cells were treated with linsitinib (1 μM) and dasatinib (100 nM) in combination or LL28 (1 μM) for 3 days. **d** Cell viability was determined by the MTT assay. **e** Anchorage-dependent colony forming ability was determined as described in the Methods section. **f** The chromatin condensation in the nuclei was observed by fluorescence microscopy, photographed, and counted. **g** The expression of cleaved PARP (Cl-PARP) was determined by Western blot analysis. **h** The cell cycle distribution in each test group was analyzed by flow cytometry. The bars represent the means ± SD; *P < 0.05, **P < 0.01, and ***P < 0.001, as determined by a two-sided Student’s *t*-test. Con: control; L + D: linsitinib and dasatinib in combination

We next assessed whether LL28 could induce apoptosis in NSCLC cells. We found that treatment with LL28 displayed dose-dependent increases in the chromatin condensation (Fig. 4a), poly (ADP-ribose) polymerase (PARP) cleavage (Fig. 4b), and the accumulation of cells at the sub-G1 phase (Fig. 4c), indicators of apoptosis, in NSCLC cells. We then assessed treatment with agents that individually target IGFIR or Src will have similar or improved efficacy compared to the LL28 treatment. To this end, we compared A549 and H460 cells treated with both linsitinib (1 μM) and dasatinib (100 nM) in combination and those treated with LL28 alone. A549 and H460 cells treated with LL28 (1 μM) showed significantly decreased viability (Fig. 4d) and colony-forming ability (Fig. 4e) along with increased apoptotic cell death (Fig. 4f-h) compared to those co-treated with linstinib and dasatinib. These findings suggested that co-targeting of IGF1R and Src by the LL28 treatment may have improved efficacy compared with that by the combinatorial treatment with linsitinib and dasatinib.

### LL28 displays minimal toxicity in vivo

**Fig. 5:**
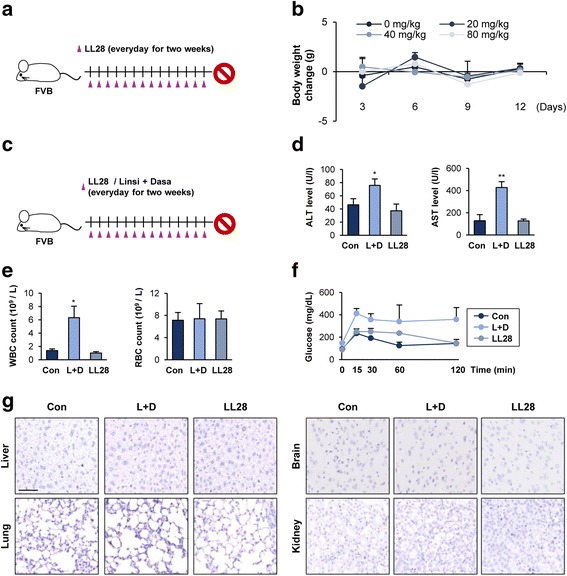
Minimal toxicity of LL28 in vivo. **a** and **c** Schematic diagram of the experiment. **b** Changes in body weight induced by treatment with LL28 in mice. **d** and **e** The level of ALT and AST in the serum and the number of WBC and RBC in whole blood obtained from mice treated with either linsitinib and dasatinib in combination or LL28 were determined as described in the Methods section. **f** The blood glucose level was determined by using tail snip blood with blood glucose test strips. **g** The histopathological changes in liver, lung, brain, and kidney from mice treated with either linsitinib and dasatinib in combination or LL28 were evaluated by using H&E-stained section of the tissues. The representative images of H&E-stained  section of the tissues were shown. Scale bar: 20 μm. The bars represent the means ± SD; *P < 0.05 and **P < 0.01, as determined by a two-sided Student’s *t*-test. Con: control; L + D: linsitinib and dasatinib in combination

We next evaluated the toxicity of LL28. To this end, several doses of LL28 (20, 40, and 80 mg/kg) were administered to mice for two weeks, and the serum levels of glutamate pyruvate transaminase (GPT), blood urea nitrogen (BUN), and creatinine were evaluated (Fig. 5a). The levels of these indicators in the serum collected from LL28-treated mice were within the reference ranges (Additional file 5: Table S4). In addition, changes in the body weight of LL28-treated mice were minimal compared with those of control mice (Fig. 5b). These results indicate that LL28 has minimal toxicity to mice.

We then compared the safety profiles of co-targeting IGFIR and Src by the LL28 treatment and those by combined treatment with dasatinib and linsitinib (Fig. 5c). To this end, we analyzed alanine aminotransferase (ALT) and aspartate aminotransferase (AST) levels (as markers of liver function) and white blood cell (WBC) and red blood cell (RBC) counts (as markers of hematological toxicities) in mice treated with linsitinib and dasatinib in combination and those treated with LL28. We found that the mice administered with linsitinib and dasatinib showed significant increases in ALT, AST, and WBC compared with vehicle-treated control mice, while these markers in the LL28-administered mice remained the same (Fig. 5d and e). We next performed glucose tolerance test as a marker of glucose homeostasis. We found obviously delayed glucose clearance in the mice treated with linsitinib and dasatinib while LL28-treated mice showed minor changes in the glucose clearance (Fig. 5f). We also histologically analyzed H&E-stained tissues from various major organs, including lung, liver, kidney, and brain, and found no detectable changes in these organs from the LL28-treated mice (Fig. 5g). Together, these data indicate that LL28 has improved safety profiles compared with the combined treatment with linsitinib and dasatinib.

### LL28 displays significant inhibitory effects on tumor growth and mutant *Kras*-driven lung tumorigenesis in vivo

**Fig. 6:**
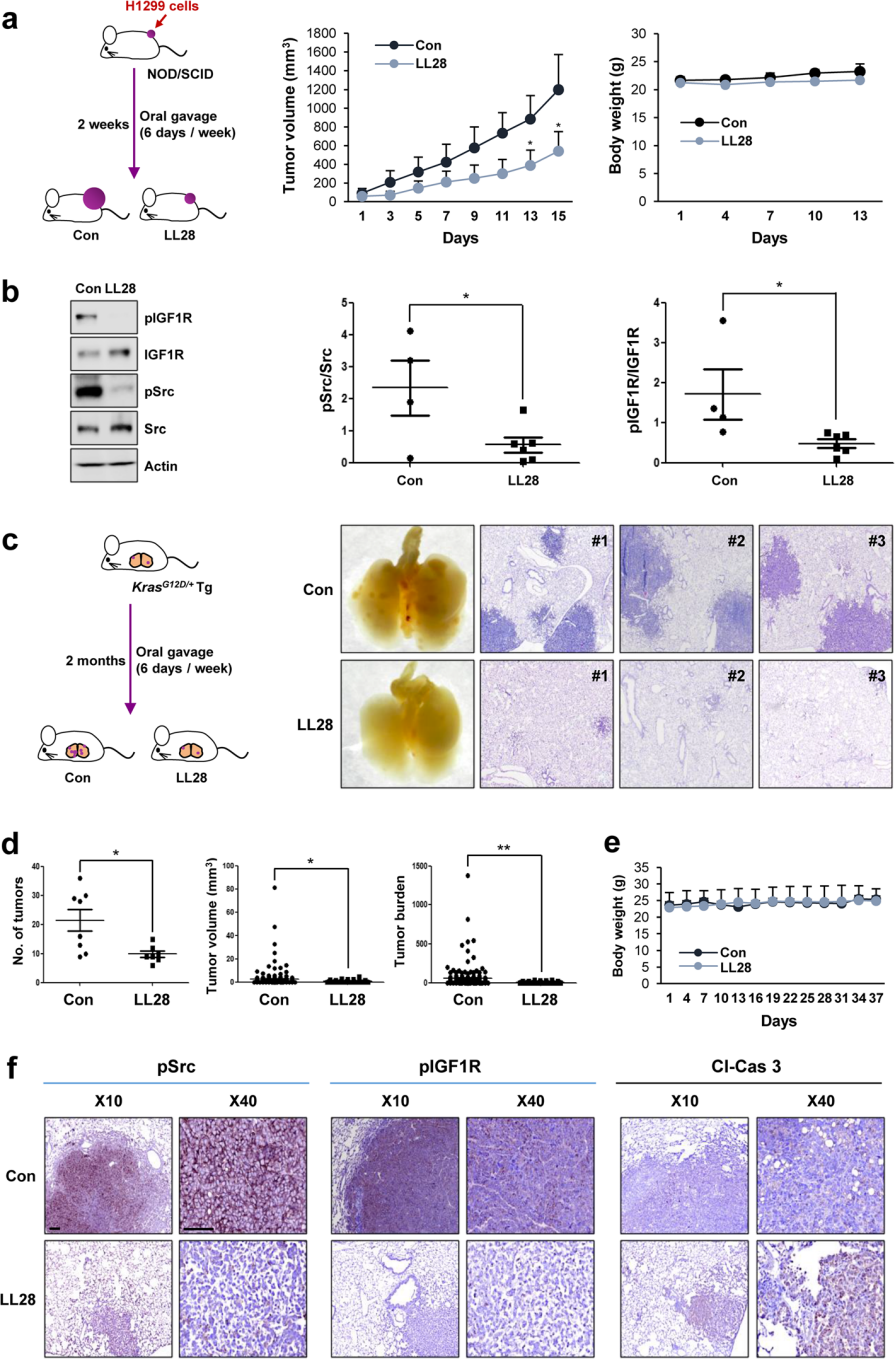
Inhibitory effects of LL28 on the growth of xenograft tumors and spontaneous lung tumorigenesis. **a** *Left.* Schematic diagram of the experiment using the tumor xenograft model. *Middle*. Antitumor effect of LL28 in vivo. *Right*. Body weight changes in vehicle- or LL28-treated mice. **b** The expression of the total and phosphorylated forms of IGF1R and Src was determined by Western blot analysis. *Left*. Representative results of the total and phosphorylated forms of IGF1R and Src. Actin blots serve as a loading control. *Right*. Densitometric analysis of the phosphorylated form of IGF1R or Src by comparison with the total expression of the corresponding protein was performed using ImageJ software. **c** *Left*. Schematic diagram of the experiment using the *Kras*^*G12D/+*^-driven spontaneous lung tumorigenesis model. *Right*. Representative images of the lungs (left) and H&E-stained section of the lungs (right) from vehicle or LL28-treated mice. **d** Microscopic analysis of H&E-stained lung tissues from vehicle- or LL28-treated *Kras*^*G12D/+*^ transgenic mice for lung tumor multiplicity and tumor volume. **e** Body weight changes in vehicle- or LL28-treated mice. **f** IHC analyses for evaluating the expression of phosphorylated forms of IGF1R and Src and cleaved caspase 3 (Cl-Cas 3) in the tumors of the lungs from vehicle- or LL28-treated mice. Scale bar: 20 μm. The bars represent the means ± SD; *P < 0.05 and **P < 0.01, as determined by a two-sided Student’s *t*-test. Con: control

We evaluated the antitumor effect of LL28 in a tumor xenograft model. Consistent with the in vitro results, treatment with LL28 significantly suppressed the growth of xenograft tumors (Fig. 6a, middle) with negligible changes in body weight (Fig. 6a, right). The expression of phosphorylated IGF1R and Src in tumors was also significantly decreased by treatment with LL28 (Fig. 6b), suggesting that LL28 exerts an antitumor effect by inhibiting tumoral IGF1R and Src activation.

It is known that Kirsten rat sarcoma viral oncogene homolog (KRAS) mutation is one of the most common genetic alterations involved in lung cancer [33] and is associated with reduced response to IGF1R-targeted therapy in lung cancer cells [34]. Moreover, in pancreatic cancer, KRAS activates Src in a pseudopodium-enriched atypical kinase 1 (PEAK1)-dependent manner or directly cooperates with Src, leading to metastatic tumor growth, therapy resistance, and accelerated tumorigenesis [35, 36]. Thus, Src appears to exert mutant *KRAS*-driven events as its downstream effector, and considering our previous finding demonstrating the involvement of IGF1R activation in the development of lung cancer [37], co-targeting IGF1R and Src may halt the progression of mutant KRAS-driven lung tumor development. Therefore, we ultimately investigated the effect of LL28 on mutant *Kras*-driven lung tumor formation. Mice were administered with vehicle or LL28 for two months (Fig. 6c). Gross and microscopic evaluation of the tumor formation in the lungs revealed that LL28 displayed a remarkable inhibitory effect on tumor formation in mice (Fig. 6c and d). In line with previous findings, the body weight of LL28-treated mice did not change compared with that of vehicle-treated mice during treatment (Fig. 6e). In addition, an immunohistochemistry (IHC) analysis further revealed that phosphorylated IGF1R and Src expression in lung tumors was significantly reduced by treatment with LL28, whereas cleaved caspase 3 expression was elevated in lung tumors from LL28-treated mice (Fig. 6f), indicating that LL28 suppresses mutant *Kras*-driven lung tumor development by inhibiting activation of both IGF1R and Src and inducing apoptosis.

## Discussion

**Fig. 7:**
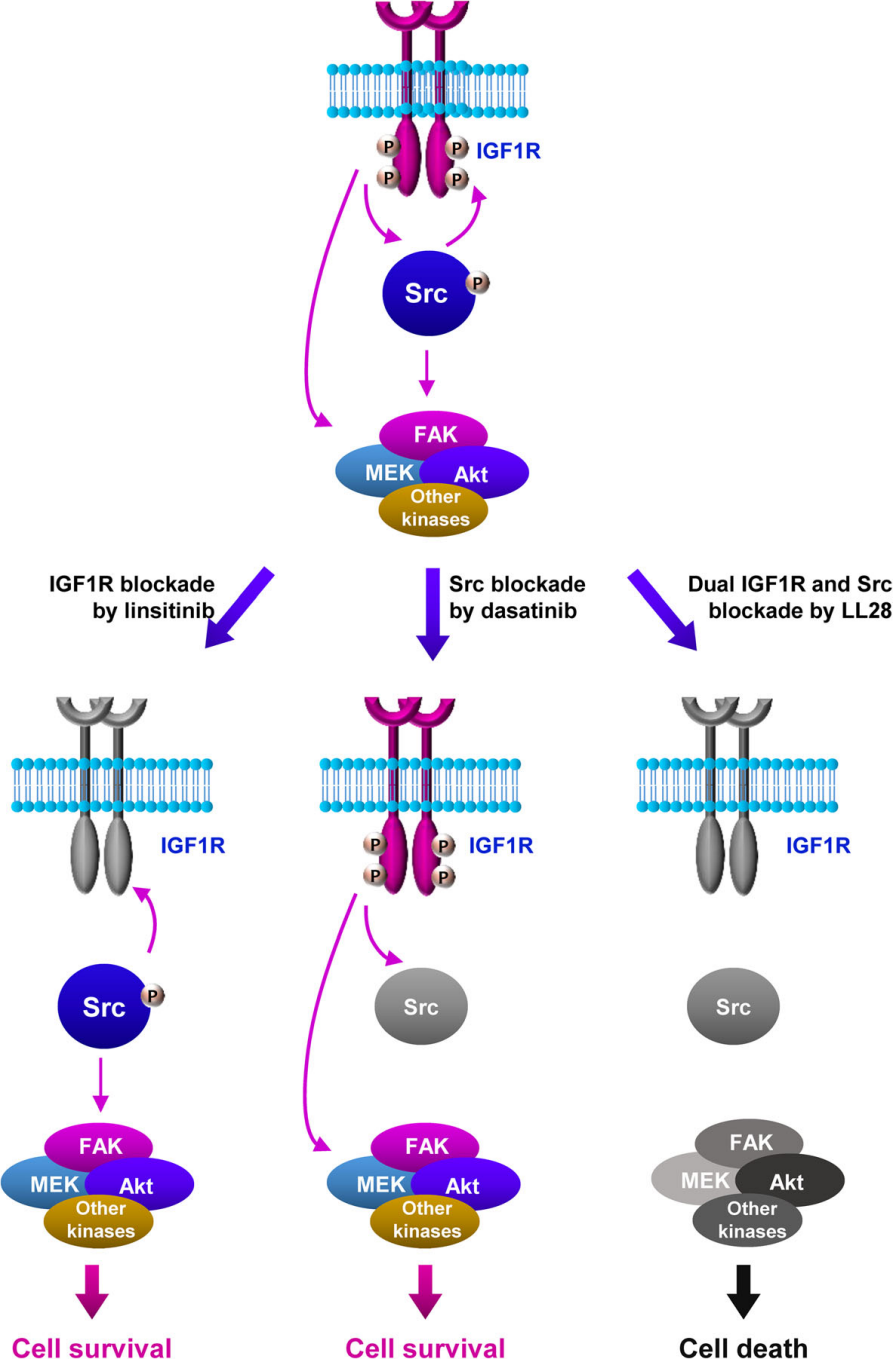
Schematic diagram to describe the action of LL28. In NSCLC cells, IGF1R and Src mutually activate in a normal state. Blockade of IGF1R by treatment with linsitinib induces the Src-mediated compensation of the IGF1R blockade. Similarly, treatment with dasatinib leads to the IGF1R-mediated compensation of the Src blockade. These overall processes cause drug resistance and maintain cell survival. LL28-induced simultaneous suppression of both IGF1R and Src blocks these feedback activations, thereby inducing apoptotic cell death in NSCLC cells

Previous studies have shown the plasticity of cancer cells, in which the blockade of particular pathways results in the activation of bypass signaling pathways, leading to cancer cells’ adaptive survival and anticancer drug resistance. We have previously demonstrated the role of Src, which functions as a shared downstream signaling unit of multiple membrane-associated growth factor receptors, in resistance to IGF1R inhibitors [17, 19]. We have devoted extensive efforts to develop potent molecularly targeted anticancer drugs blocking both IGF1R and Src. The studies reported herein demonstrate that a 4-aminopyrazolo[3,4-*d*]pyrimidine-based dual IGF1R/Src inhibitor, LL28, has promising anticancer activity in vitro and in vivo with minimal toxicity. LL28 effectively suppressed IGF1R and Src signaling and NSCLC cell viability and colony forming ability in vitro. Furthermore, LL28 significantly suppressed the growth of xenograft tumors and mutant *Kras*-driven lung tumorigenesis with minimal toxicity in vivo. Our results provide preclinical evidence for the use of LL28 as a dual IGF1R/Src-targeting drug in cancer therapy (Fig. 7).

IGF1R signaling has been shown to play a crucial role in the development and progression of several types of cancer, including lung cancer [37, 38]. Indeed, several preclinical and early phase clinical trials demonstrated the effectiveness of IGF1R-targeted therapies as a monotherapy or a combinatorial therapy with other anticancer drugs [39]. However, the currently available IGF1R-targeted therapies have shown marginal efficacy and toxicity in advanced clinical trials, presumably due to the activation of bypass signaling as a resistance mechanism. Emergence of drug resistance through activation of compensatory signaling pathways seemed to cause these failures [39]. Multifactorial diseases, such as Alzheimer’s disease, cancer, diabetes, and immunoinflammatory disorders, are highly heterogeneous [40]. Various clinical trials employing single-target therapies in such diseases have resulted in drug resistance through redundancy and crosstalk between different signaling pathways, suggesting the necessity of combinatorial therapy by combining drugs in different therapeutic classes [40]. Single-targeted drugs have shown particularly poor efficacy in a highly heterogeneous malignancy such as NSCLC [41]. Therefore, it is of considerable importance to obtain alternative strategies to target the IGF1R signaling while preventing the emergence of drug resistance.

Both IGF1R and Src are frequently overexpressed in various types of human cancer, inversely correlated with patient survival, and known to crosstalk with each other [19, 38, 42]. We have previously demonstrated the crucial role of Src in resistance to IGF1R-targeted anticancer drugs [17, 19, 21]. In these studies, combined treatment with an IGF1R inhibitor and a Src inhibitor showed significantly enhanced anticancer effects compared with treatment with each drug alone. These findings led us to hypothesize that combinatorial strategies of IGF1R and Src inhibitors would be an effective anticancer strategy in overcoming drug resistance. However, several side effects and toxicities, such as hematological toxicities and diabetes-like symptoms (e.g., hyperglycemia and hyperinsulinemia) in the case of IGF1R-targeted therapies and hematological toxicities (e.g., leukopenia, neutropenia, and thrombocytopenia) in the case of Src inhibitors, have been reported in recent clinical trials utilizing these drugs [43–45]. Hence, the main drawbacks of the combinatorial strategies could be the enhanced toxicities from the concurrent use of two inhibitors. In fact, co-formulation of two and more drugs in a single dosage is frequently accompanied by severe side effects due to the highly complex pharmacokinetics (PK) and pharmacodynamics (PD), and drug cocktails usually result in poor patient compliance [46]. Accordingly, single compounds with multiple biological activities have been highlighted as an innovative drug development strategy to minimize potential toxicity [47]. Hence, we hypothesized that developing dual kinase inhibitors concurrently targeting IGF1R and Src would be beneficial. Recent studies proposed oxadiazinones and oxoacetohydrazides as scaffolds for dual IGF1R/Src inhibitors [21, 22], but developing additional chemical entities and evaluating them using multiple in vitro and in vivo experimental models should be required because of the importance of co-targeting IGF1R and Src for anticancer therapy. Our results from several in vitro and in vivo biological analyses shown in the current study demonstrate that LL28, a novel 4-aminopyrazolo[3,4-*d*]pyrimidine-based drug, offers reasonable efficacy and a good safety profile as a dual IGF1R/Src inhibitor. LL28 exhibited similar inhibitory effects on cell viability across a panel of NSCLC cells with various histological and genetic backgrounds. We recently observed that IGF1R and Src were co-activated in several lung cancer cell lines and a tissue microarray consisting of lung adenocarcinoma and squamous cell carcinoma [19]. Hence, the similar potency of LL28 in NSCLC cells regardless of genetic alterations and histology could be due to the coactivation of IGF1R and Src. Notably, LL28 effectively suppressed the colony forming abilities of NSCLC under anchorage-dependent conditions. Clonogenicity under adherent conditions is correlated with the ability of cells to survive without cell-to-cell interactions [32]; thus, these results raise the possibility that LL28 may be effective cancer therapeutic agent in patients with metastatic dissemination. Additional evaluation of LL28 using relevant experimental models should be the focus of further studies.

## Conclusions

Our findings provide evidence that LL28 effectively disrupts IGF1R and Src signaling simultaneously and significantly inhibits the viability and colony forming ability of NSCLC cells in vitro. Moreover, when tested in vivo in animal models, LL28 significantly suppresses the growth of xenograft tumors and mutant *Kras*-driven lung tumorigenesis. The excellent safety profiles of LL28 shown in mice further highlights its potential as a clinically relevant anticancer drug. These findings suggest that LL28 is a potential lead candidate to develop anticancer drugs targeting both IGF1R and Src. The knowledge obtained from these studies may be incorporated to design better anticancer agents. Further studies are necessary to evaluate the efficacy of LL28 in additional preclinical and clinical settings.

## Methods

### Cell culture

Human lung cancer cell lines (A549, H1299, H460, H2170, H2122, H1944, HCC15, H596, H522, H1993, H226B, and H226Br) and a breast cancer cell line (MCF7) were purchased from the American Type Culture Collection (ATCC, Manassas, VA, USA) or kindly provided by Dr. John V. Heymach (MD Anderson Cancer Center, Houston, TX, USA). R- cells were kindly provided by Dr. Renato Baserga (Columbia University, NY, USA). Lung cancer cells were cultured in RPMI 1640 medium supplemented with 10% fetal bovine serum (FBS) and antibiotics (all from Welgene, Daegu, Republic of Korea). MCF7 and R- cells were maintained in DMEM (Welgene) supplemented with 10% FBS and antibiotics. Cells were maintained at 37 °C in a humidified atmosphere with 5% CO_2_ and subcultured once or twice a week. Lung cancer cell lines and MCF7 cells were authenticated and validated using the AmplFLSTR identifier PCR Amplification Kit (Applied Biosystems, Foster City, CA, USA; cat. No. 4322288) in 2013 and 2016. Cells that had been passaged for fewer than 6 months after receipt or resuscitation of validated cells were used in this study.

### Reagents

Antibodies against pIGF1R (Y1135/6), IGF1R, pSrc (Y416), Src, phosphor-tyrosine (pTyr), pMEK1/2, MEK1/2, pAkt (S473), Akt, and cleaved caspase 3 were purchased from Cell Signaling Technology (Danvers, MA, USA). Antibodies against cleaved PARP and FAK  were purchased from BD Biosciences (San Jose, CA, USA). A primary antibody against pFAK (Y576/577) was purchased from Thermo Fisher Scientific (Waltham, MA, USA). Primary antibodies against IGF1R, IR, and actin and the horseradish peroxidase (HRP)-conjugated anti-mouse secondary antibody were purchased from Santa Cruz Biotechnology (Santa Cruz, CA, USA). HRP-conjugated anti-rabbit  and anti-goat secondary antibodies were purchased from GeneTex (Irvine, CA, USA). Linsitinib and dasatinib were purchased from Selleckchem (Houston, TX, USA) or LC Laboratories (Woburn, MA, USA). 3-(4,5-Dimethylthiazol-2-yl)-2,5-diphenyl tetrazolium bromide (MTT) and other chemicals were purchased from Sigma-Aldrich (St. Louis, MO, USA) unless otherwise specified.

### MTT assay

Cells were seeded into 96-well multiwall plates at a density of 1-2 × 10^3^ cells/well and allowed to attach for 24 h. Cells were treated with vehicle or the indicated concentrations of LL28 diluted in complete media for 3 days. Cells were further incubated with the MTT solution (final concentration of 500 μg/ml) for 4 h at 37 °C. The formazan products were dissolved in dimethylsulfoxide (DMSO), and the absorbance was measured at 570 nm. The data are presented as a percentage of the control group.

### Anchorage-dependent colony formation assay

Cells were seeded into 6-well plates at a density of 300 cells/well and treated with various concentrations of LL28 for two weeks. The drug-containing medium was changed once or twice a week. Colonies were fixed with 100% methanol, stained with 0.002% crystal violet solution, and washed with deionized water several times. The colonies were imaged and counted using ImageJ software (National Institutes of Health, Bethesda, MA, USA).

### Anchorage-independent colony formation assay

Cells were mixed with sterile 1% agar solution (final concentration of 0.4%) and poured onto 1% base agar in 24-well plates. LL28 diluted in complete medium was added to the agar after solidification of the top agar. Cells embedded in the top agar were incubated for 2 weeks at 37 °C with 5% CO_2_. The medium was changed twice a week. After incubation, the colonies were stained with MTT solution, imaged and counted.

### Western blot analysis

Cells were treated with LL28, linsitinib, or dasatinib for the indicated time intervals. Before harvesting, the cells were stimulated with 10% FBS for 20 min. Total cell lysates were prepared with modified radioimmunoprecipitation (RIPA) lysis buffer [50 mM Tris-HCl (pH 7.4), 150 mM NaCl, 1 mM EDTA, 0.25% sodium deoxycholate, 1% Triton X-100, protease inhibitor cocktail (Roche Applied Science, Indianapolis, IN, USA), and phosphatase inhibitor cocktail (Roche)]. Equal amounts of protein (20 μg) were subjected to 8% SDS-PAGE and electrically transferred onto polyvinylidene difluoride (PVDF) membranes (Atto Corp., Tokyo, Japan). Membranes were blocked with blocking buffer [5% non-fat dry milk in Tris-buffered saline (TBS) containing 0.01% Tween-20 (TBST)] for 1 h at room temperature. The membranes were incubated with primary antibodies diluted in 3% BSA in TBST (1: 1000) overnight at 4 °C, were washed multiple times with TBST, and were incubated with secondary antibodies diluted in 5% non-fat dry milk in TBST (1:5000) for 1 h at room temperature. The membranes were washed multiple times with TBST and visualized using an enhanced chemiluminescence (ECL) detection kit (Thermo Fisher Scientific). Densitometric analysis was performed using ImageJ software.

### Hoechst 33,342 staining

Cells were treated with LL28 (0.1 and 1 μM) or linsitinib (1 μM) and dasatinib (100 nM) in combination for 3 days. These cells were stained with the Hoechst 33,342 solution (final 10 μg/ml) at 37 °C for 20 min. Stained cells were observed under an inverted fluorescence microscope (EVOS FL Cell Imaging System; Thermo Fisher Scientific) and photographed.

### Cell cycle analysis

A549, H1299, and H460 cells were treated with increasing concentrations of test drugs (LL28 or linsitinib and dasatinib in combination) for 3 days. Adherent and floating cells were collected and washed with PBS. Cells were fixed with 100% methanol and stained with a 50 μg/ml propidium iodide (PI) solution containing 50 μg/ml RNase A for 30 min at room temperature. Fluorescence intensity was analyzed by flow cytometry using a FACSCalibur® flow cytometer (BD Biosciences). Cell cycle analysis was performed using CellQuest software (BD Biosciences).

### Animal experiments

All animal experiments were performed according to protocols approved by the Seoul National University Institutional Animal Care and Use Committee. Mice were fed standard mouse chow and water *ad libitum* and housed in temperature- and humidity-controlled facilities with a 12-h light/12-h dark cycle. For xenograft experiments, H1299 cells (diluted in equal amount of PBS and Matrigel) were subcutaneously injected into the right flank of 6-week-old female Non-Obese Diabetic-Severe Combined Immunodeficiency (NOD/SCID) mice. After the tumor volume reached 50-150 mm^3^, the mice were randomly grouped and administered with vehicle (10% DMSO in corn oil) or LL28 (80 mg/kg) 6 days per week for 2 weeks. Tumor growth was determined by measuring the short and long diameter of the tumor with a caliper, and body weight was measured twice per week to monitor toxicity. In addition, to evaluate the effect of LL28 on mutant *KRAS*-driven lung tumorigenesis, two-month-old *Kras*^*G12D/+*^ transgenic mice [48] were randomized and treated with vehicle or LL28 (80 mg/kg) for 8 weeks. The mice were euthanized, and tumor formation was evaluated and compared with that of the vehicle-treated control group. Microscopic evaluations of lung tissue were also performed to measure mean tumor number (N) and volume (V) in a blinded fashion after hematoxylin and eosin (H&E) staining. The number and size of tumors were calculated in five sections uniformly distributed throughout each lung. In both animal experiments, the tumor volume was calculated using the following formula: tumor volume (mm^3^) = (short diameter)^2^ × (long diameter) × 0.5.

### Toxicity test

FVB mice were treated with vehicle, LL28 (20, 40, and 80 mg/kg), or linsitinib (25 mg/kg, dissolved in 25 mM tartaric acid solution) and dasatinib (20 mg/kg, dissolved in 80 mM citric acid solution) in combination every day for 2 weeks. Blood was collected from euthanized mice under isoflurane-induced deep anesthesia by cardiac puncture. After allowing blood coagulation at 4 °C, serum was collected by centrifugation at 3000 rpm for 10 min at 4 °C. Analysis of the level of BUN, creatinine, GPT, ALT, and AST in the serum was performed using a veterinary hematology analyzer (Fuji DRI-Chem 3500 s, Fujifilm, Tokyo, Japan) according to the manufacturer’s provided protocols. The number of WBC and RBC in whole blood was measured with an automatic hematology analyzer (Advia 2120i, Siemens, Germany) according to the manufacturer’s provided protocols. The histopathological changes in liver, lung, brain, and kidney were evaluated by using H&E-stained section of the tissues.

### Glucose tolerance test

Glucose tolerance test was performed as described in the previously published literature [49]. Briefly, after fasting for a day, mice were intraperitoneally administered with 10% glucose solution (100 μl/20 g mouse). The blood glucose levels in mice at various time intervals were determined by using tail snip blood with blood glucose test strips.

### Immunohistochemistry

Sections derived from formalin-fixed and paraffin-embedded murine lung tissues were deparaffinized by incubation overnight at 65 °C followed by rehydration in sequential xylene and ethanol rinses. After incubation with hydrogen peroxide, the slides were washed with PBS and then incubated with 0.4% Triton X-100. The sections were incubated with blocking solution (Dako Protein Block, Dako, Glostrup, Denmark) for 30 min at room temperature after washing with PBS. The sections were further incubated with primary antibodies (phosphorylated IGF1R, phosphorylated Src, and cleaved caspase 3 [all from Cell Signaling], diluted at 1:200) overnight at 4 °C, washed with PBS several times, incubated with the corresponding biotinylated secondary antibodies (diluted at 1:500), and then washed with PBS multiple times. After adding avidin-biotin complexes (Vector Laboratories), the sections were visualized using diaminobenzidine (DAB) detection reagent (Enzo Life Sciences, Farmingdale, NY, USA) and mounted with a mounting solution (Vector Laboratories, Burlingame, CA, USA).

### Synthesis of 3a-f and 4a-d

#### General information

Unless otherwise specified, all reagents and solvents were purchased from commercial suppliers and used without further purification. All reactions were monitored by thin-layer chromatography (TLC) on precoated silica plates 60 F_254_ (Merck, Darmstadt, Germany). Column chromatography was carried out on Zeochem silica gel (Zeo prep 60, 40-63 μm; Zeochem, Lake Zurich, Switzerland). ^1^H nuclear magnetic resonance (NMR) (300, 400, 500, and 600 MHz) and ^13^C NMR (100, 125, and 150 MHz) spectra were recorded on GEMINI 2000 (VARIAN, Palo Alto, CA, USA), JNM-LA300 (JEOL, Tokyo, Japan) or AVANCE 400 (Bruker, Billerica, MA, USA). Chemical shifts (δ) were reported in parts per million (ppm) and were referenced to the residual solvent peak. Coupling constants (*J*) were reported in hertz (Hz). All electrospray ionization mass spectrometry (ESI-MS) was measured on a 6130 Single Quadrupole liquid chromatography/mass spectrometry (LC/MS) (Agilent Technologies, CA, USA). High-resolution mass spectra (HRMS) were acquired under fast atom bombardments (FAB) conditions on a JMS-700 MStation (JEOL, Germany).

### Synthesis

The details of the synthesis and characterizations of all compounds are described in the supplementary information. Representative procedures for the synthesis of **3a**-**f** and **4a**-**d** are as follows.

### N^2^-(3-((3-(4-(4-amino-1-isopropyl-1H-pyrazolo[3,4-d]pyrimidin-3-yl)phenyl)prop-2-yn-1-yl)oxy)-4-methoxyphenyl)-N^4^-(quinolin-3-yl)pyrimidine-2,4-diamine (4b)

Substrate **2a** (100 mg, 0.26 mmol), Pd(PPh_3_)_2_Cl_2_ (7.4 mg, 0.01 mmol), and CuI (1.5 mg, 0.008 mmol) were first added to a two-necked flask under a N_2_ atmosphere, which was followed by the addition of anhydrous dimethylformamide (DMF) (5 mL), alkyne **24e** (115 mg, 0.29 mmol), and triethylamine (TEA) (0.37 mL, 2.64 mmol). The reaction flask was covered with aluminum foil, and the mixture was stirred for 3 h at room temperature. Distilled water was added, and the resultant mixture was extracted with ethyl acetate (3 X 200 mL). The organic phase was dried with anhydrous sodium sulfate, the solvent was evaporated, and the crude product was purified using silica gel chromatography with ethyl acetate/methanol gradient to afford a light-yellow solid (121 mg, 0.19 mmol, 72.0% yield). ^1^H NMR (600 MHz, DMSO-d6) δ 9.79 (s, 1H), 9.14 (s, 1H), 8.94 (brs, 1H), 8.89 (s, 1H), 8.24 (s, 1H), 8.08 (d, *J* = 5.9 Hz, 1H), 7.92 (d, *J* = 8.3 Hz, 1H), 7.76 (brs, 1H), 7.63 (d, *J* = 8.2 Hz, 2H), 7.59-7.53 (m, 3H), 7.51 (d, *J* = 7.8 Hz, 2H), 7.31 (d, *J* = 7.3 Hz, 1H), 6.96 (d, *J* = 8.8 Hz, 1H), 6.30 (d, *J* = 5.5 Hz, 1H), 5.06 (sep, 1H), 4.94 (s, 2H), 3.81 (s, 3H), 1.48 (d, *J* = 6.4 Hz, 6H) ppm. ^13^C NMR (150 MHz, DMSO-d6) δ 160.38, 159.88, 158.06, 156.41, 155.42, 153.45, 146.51, 144.99, 144.73, 143.21, 142.38, 134.03, 133.39, 132.08 (3C), 128.46, 128.32 (2C), 128.17, 127.29, 126.89, 126.73, 121.47, 121.06, 113.91, 112.58, 108.70, 98.86, 97.39, 86.15, 86.11, 57.00, 55.92, 48.16, 21.71 (2C) ppm. LC-MS (ESI) m/z 649.00 [M + H]^+^. HRMS (FAB) calculated for C_37_H_32_N_10_O_2_ [M + H]^+^: 649.2788, found: 649.2795.

### Statistical analysis

The data are presented as the means ± SD. All in vitro experiments were independently performed at least twice, and a representative result is presented. The data were calculated or analyzed with Microsoft Excel software (Microsoft Corp., Redmond, MA, USA). The IC_50_ values were determined by non-linear regression analysis using Graphpad Prism 5 (GraphPad Software, Inc., La Jolla, CA, USA). Statistical significance was determined using a two-sided Student’s t-test. A *P* value of less than 0.05 was considered significant.

## Additional files

Additional file 1: Synthesis and characterization of compounds **3a-f**, **4a-d** and their intermediates. (PDF 2667 kb)

Additional file 2: Table S1. The IC_50_ values showing the inhibitory effect of selected compounds. (PDF 201 kb)

Additional file 3: Table S2. The IC_50_ values showing the inhibitory effect of LL28 on the viability of a panel of human lung cancer cells. (PDF 177 kb)

Additional file 4: Table S3. The IC_50_ values showing the inhibitory effect of LL28 on the anchorage-dependent colony -forming ability of a panel of human lung cancer cells. (PDF 176 kb)

Additional file 5: Table S4. Changes in the level of GPT, BUN, and creatinine by treatment with LL28 in mice. (PDF 109 kb)
