# Correction: Development of a 4-aminopyrazolo[3,4-*d*]pyrimidine-based dual IGF1R/Src inhibitor as a novel anticancer agent with minimal toxicity

**DOI:** 10.1186/s12943-023-01754-6

**Published:** 2023-03-08

**Authors:** Ho Jin Lee, Phuong Chi Pham, Seung Yeob Hyun, Byungyeob Baek, Byungjin Kim, Yunha Kim, Hye-Young Min, Jeeyeon Lee, Ho-Young Lee

**Affiliations:** 1 Creative Research Initiative Center for Concurrent Control of Emphysema and Lung Cancer, College of Pharmacy, Seoul National University, Seoul, 08826 Republic of Korea; 2 College of Pharmacy and Research Institute of Pharmaceutical Sciences, Seoul National University, Seoul, 08826 Republic of Korea; 3 Department of Molecular Medicine and Biopharmaceutical Science, Graduate School of Convergence Science and Technology, and College of Pharmacy, Seoul National University, Seoul, 08826 Republic of Korea

**Correction: Mol Cancer 17, 50 (2018)**

**https://doi.org/10.1186/s12943-018-0802-4**

In our publication in Molecular Cancer entitled ‘Development of a 4-aminopyrazolo[3,4-d]pyrimidine-based dual IGF1R/Src inhibitor as a novel anticancer agent with minimal toxicity [Mol Cancer 17, 50 (2018); doi: 10.1186/s12943-018-0802-4]’ [1], we regret the errors in Fig. 2b in the printed version. In detail, the western blot images in Fig. 2b (Src blots in the A549 group and Actin blots in the H1299 group) were inadvertently placed by mistake. We have double-checked the original data and found that the inadvertent errors occurred during image assembling. The corrected Fig. 2 is given here, and this correction does not change the scientific conclusions of the article.

**Fig. 2:**
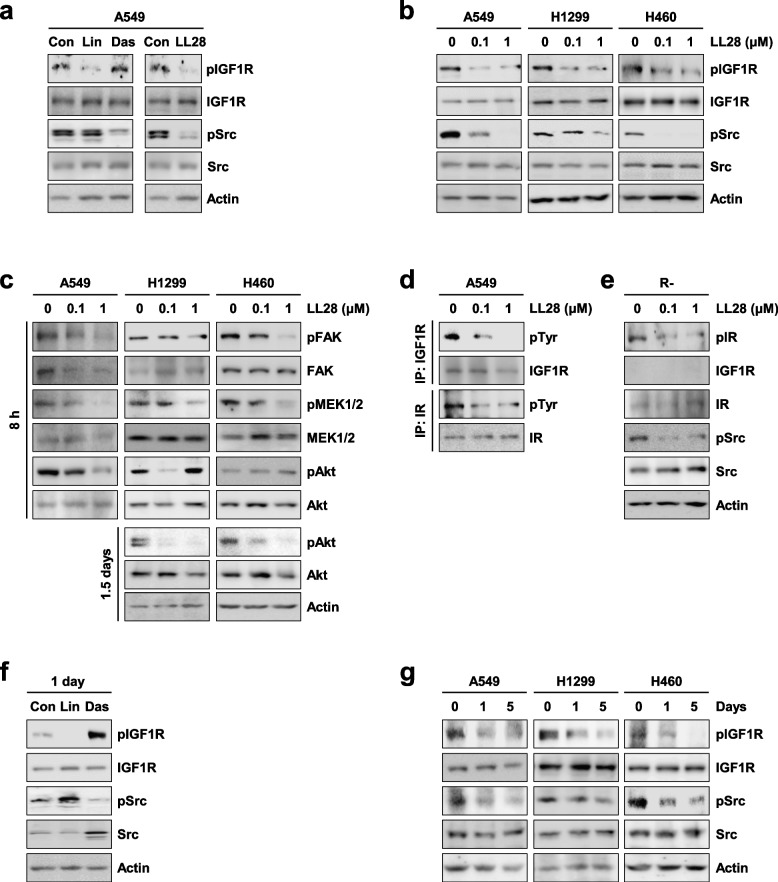
Inhibitory effect of LL28 on the activation of both IGF1R and Src. **a** A549 cells were treated with linsitinib (1 μM), dasatinib (100 nM), or LL28 (1 μM) for 4 h. Before harvesting, cells were stimulated with FBS for 20 min. The expression of total and phosphorylated IGF1R and Src was evaluated by Western blot analysis. **b** and **c** A549, H1299, and H460 cells were treated with LL28 (0.1 and 1 μM) for 8 h (**b** and **c**) or 1.5 days (**c**). **b** The expression of total and phosphorylated IGF1R and Src was evaluated by Western blot analysis. **c** The expression of the total and phosphorylated forms of several kinases was evaluated by Western blot analysis. **d** Total cell lysates of A549 cells treated with LL28 for 8 h were immunoprecipitated with anti-IGF1R or anti-IR antibodies. The immunoprecipitants were further subjected to Western blot analysis using anti-pTyr, anti-IGF1R, and anti-IR antibodies. **e** R- cells were treated with LL28 (0.1 and 1 μM) for 8 h. The expression of total and phosphorylated IGF1R and Src was determined by Western blot analysis. **f** A549 cells were treated with linsitinib (1 μM) or dasatinib (100 nM) for 1 day. The expression of total and phosphorylated IGF1R and Src was evaluated by Western blot analysis. **g** A549, H1299, and H460 cells were treated with LL28 (0.1 μM) for 1, 3, and 5 days. The expression of total and phosphorylated IGF1R and Src was evaluated by Western blot analysis. Con: control; Lin: linsitinib; Das: dasatinib

We sincerely apologize for any inconveniences these mistakes may have caused.
