# Supplementary material for: Development of a 4-aminopyrazolo[3,4-d]pyrimidine-based dual IGF1R/Src inhibitor as a novel anticancer agent with minimal toxicity
Source: Mol Cancer. 2018 Feb 19;17:50. doi: 10.1186/s12943-018-0802-4 (PMC5817804; doi:10.1186/s12943-018-0802-4)
Supplement: Supplementary file 1 — Synthesis and characterization of compounds 3a-f, 4a-d and their intermediates. (PDF 2667 kb) [file 12943_2018_802_MOESM1_ESM.pdf]

Additional file_01

Synthesis and characterization of compounds **3a-f**, **4a-d** and their intermediates.

I. Synthesis S2-S3

Scheme S1. Synthesis of 3-phenylpyrazolopyrimidines..

Scheme S2. Synthesis of 2,4-bis-arylamino-1,3-pyrimidines modules.

II. Characterization of 3a-f, 4a-d, and 24a-e S4-S10

III. ^{1}H and ^{13}C NMR spectra S11-S20

I. Synthesis

Synthesis of 1a-b. The 4-aminopyrazolo[3,4-*d*]pyrimidines modules **1a-b** were synthesized according to the reported procedure, with slight modifications as follows (**Scheme S1**).[1] **8a-b** reacted with malononitrile in the presence of sodium hydride in anhydrous THF to yield **9a-e**. Subsequent methylation with dimethyl sulfate in the presence of sodium bicarbonate in dioxane-water mixtures afforded **10a-b**, which were cyclized to **11a-b** by reacting with hydrazine monohydrate in ethanol. Reaction of **11a-b** with formamide at 160 °C for 5h afforded key intermediates **1a-b**.

Scheme S1. Synthesis of 3-phenylpyrazolopyrimidines.

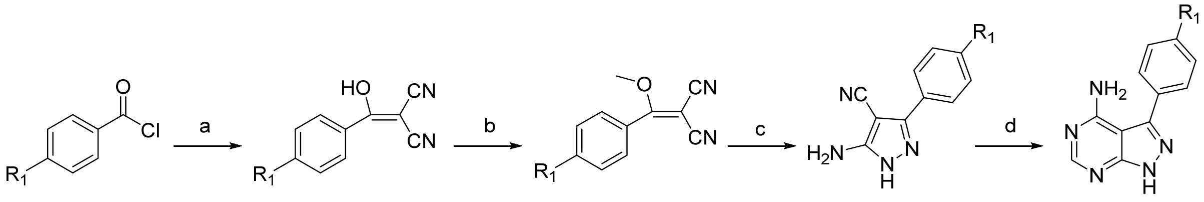

8a-b **9a-b** **10a-b** **11a-b** **12a-b (1a-b)**

8-12 a: R_1 = I, **b**: R_1 = CH_3

Reagents and conditions: a) Malononitrile, NaH, THF anhydrous, 0 °C to r.t, 1 h, 65-93%; b) Me_2SO_4, $NaHCO_3$, 1,4-dioxane:H_2O = 5:1, 80 °C, 3 h, 50-65%; c) $NH_2NH_2\cdot H_2O$, EtOH, 120 °C, 30 min, 75-83%; d) $HCONH_2$, 160 °C, 5 h, 36-83%.

Synthesis of 24a-e. The synthesis of the 2,4-bis-arylamino-1,3-pyrimidines modules was modified from the reported procedure as depicted in **Scheme S2**.[2] Reaction of 2,4-dichloropyrimidine **19** with 3-aminoquinoline **20** in the presence of DIEA produced **21**, which reacted with the aminophenol derivatives **22a-e** to form **23a-e**. *O*-alkylation of phenol with propargyl bromide under basic conditions afforded **24a-e,** respectively.

Scheme S2. Synthesis of 2,4-bis-arylamino-1,3-pyrimidines modules.

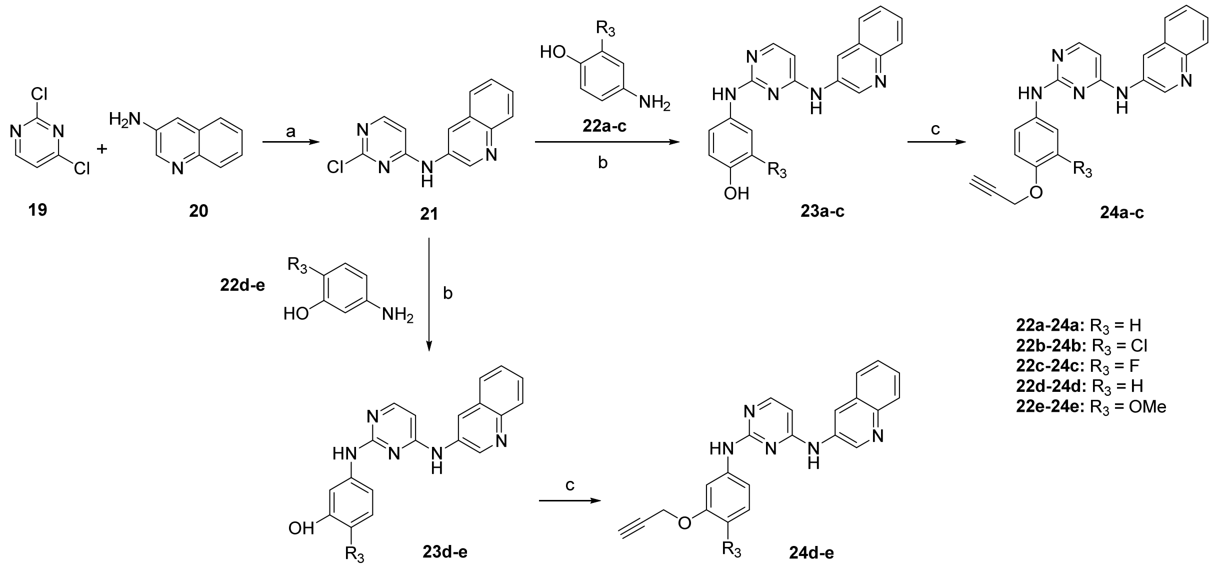

Reagents and conditions: a) DIEA, *i*-PrOH, refluxed 100 °C, 24 h, 70%; b) DMSO, 90 °C, 2 h, 88-99%; c) Propargyl bromide, Cs_2CO_3, DMF, r.t, 2 h, 59-67%.

II. Characterization of 3a-f, 4a-d, and 24a-e

N^2-(4-((3-(4-(4-amino-1-isopropyl-1H-pyrazolo[3,4-d]pyrimidin-3-yl)phenyl)prop-2-yn-1-yl)oxy)phenyl)-N^4-(quinolin-3-yl)pyrimidine-2,4-diamine (3a)

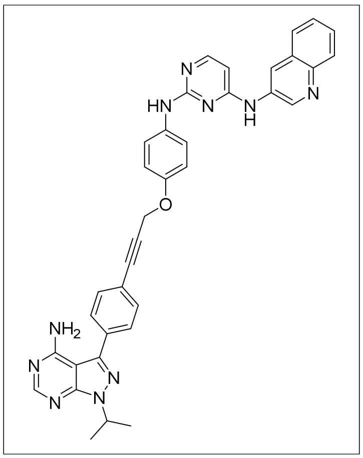

This compound was synthesized using the general procedure depicted in the method section (yield = 70.0%). ^{1}H NMR (500 MHz, DMSO-d_6) δ 9.83 (s, 1H), 9.17 (s, 1H), 8.94 (brs, 2H), 8.24 (s, 1H), 8.08 (d, J = 5.7 Hz, 1H), 7.93 (d, J = 8.4 Hz, 1H), 7.82 (d, J = 5.5 Hz, 1H), 7.67-7.65 (m, 4H), 7.59-7.55 (m, 4H), 7.02 (d, J = 8.9 Hz, 2H), 6.31 (d, J = 5.7 Hz, 1H), 5.09-5.04 (m, 3H), 1.48 (d, J = 6.7 Hz, 6H) ppm. ^{13}C NMR (125 MHz, DMSO-d_6) δ 160.43, 159.79, 158.07, 156.44, 155.44, 153.46, 152.34, 145.15, 143.31, 142.40, 134.48, 134.01, 133.47, 132.14 (3C), 128.52, 128.44 (3C), 128.18, 127.28, 126.97, 126.82, 121.48, 121.23, 115.04 (2C), 98.82, 97.42, 86.34, 86.06, 56.43, 48.18, 21.73 (2C) ppm. LC-MS (ESI) m/z 619.01 $[M + H]^+$. HRMS (FAB) calculated for $C_{36}H_{30}N_{10}O$ $[M + H]^+$: 619.2682, found: 619.2675.

N^2-(4-((3-(4-(4-amino-1-isopropyl-1H-pyrazolo[3,4-d]pyrimidin-3-yl)phenyl)prop-2-yn-1-yl)oxy)-3-chlorophenyl)-N^4-(quinolin-3-yl)pyrimidine-2,4-diamine (3b)

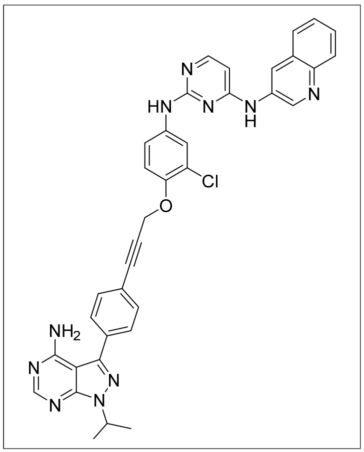

This compound was synthesized using the general procedure depicted in the method section (yield = 71%). ^{1}H NMR (600 MHz, DMSO-d_6) δ 9.87 (s, 1H), 9.33 (s, 1H), 8.97 (d, J = 2.3 Hz, 1H), 8.92 (brs, 1H), 8.24 (s, 1H), 8.13 (d, J = 5.9 Hz, 1H), 7.96 (s, 1H), 7.94 (d, J = 8.3 Hz, 1H), 7.85 (d, J = 7.8 Hz, 1H), 7.67 (d, J = 8.2 Hz, 2H), 7.64 (dd, J = 8.9 Hz, 2.6 Hz, 1H), 7.61-7.55 (m, 4H), 7.27 (d, J = 9.2 Hz, 1H), 6.36 (d, J = 5.5 Hz, 1H), 5.15 (s, 2H), 5.06 (sep, 1H), 1.48 (d, J = 6.9 Hz, 6H) ppm. ^{13}C NMR (150 MHz, DMSO-d_6) δ 160.45, 159.31, 158.05, 156.31, 155.42, 153.44, 147.23, 145.26, 143.42, 142.37, 135.60, 133.88, 133.56, 132.17 (2C), 128.49, 128.44 (2C), 128.13, 127.40, 127.07, 126.78, 121.70, 121.45, 121.31, 120.84, 118.90, 115.48, 99.43, 97.41, 86.54, 85.76, 57.59, 48.17, 21.70 (2C) ppm. LC-MS (ESI) m/z 653.00 $[M + H]^+$. HRMS (FAB) calculated for $C_{36}H_{29}ClN_{10}O$ $[M + H]^+$: 653.2293, found: 653.2297.

N[2]-(4-((3-(4-(4-amino-1-isopropyl-1H-pyrazolo[3,4-d]pyrimidin-3-yl)phenyl)prop-2-yn-1-yl)oxy)-3-fluorophenyl)-N[4]-(quinolin-3-yl)pyrimidine-2,4-diamine (3c)

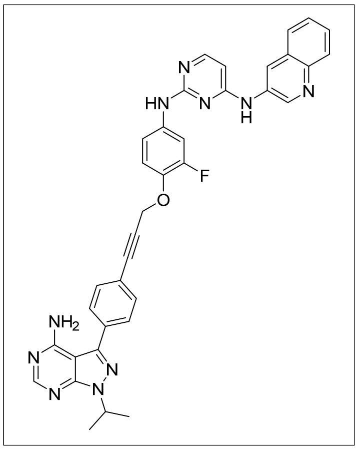

This compound was synthesized using the general procedure depicted in the method section (yield = 45.7%). ^{1}H NMR (600 MHz, DMSO-d_6) δ 9.93 (s, 1H), 9.40 (s, 1H), 8.97 (d, J = 2.7 Hz, 1H), 8.92 (brs, 1H), 8.24 (s, 1H), 8.12 (d, J = 5.5 Hz, 1H), 7.94 (d, J = 8.3 Hz, 1H), 7.86-7.82 (m, 2H), 7.67 (d, J = 8.2 Hz, 2H), 7.61-7.55 (m, 4H), 7.42 (d, J = 8.7 Hz, 1H), 7.26 (t, J = 9.4 Hz, 1H), 6.37 (d, J = 6.0 Hz, 1H), 5.11 (s, 2H), 5.06 (sep, 1H), 1.48 (d, J = 6.9 Hz, 6H) ppm ^{13}C NMR (150 MHz, DMSO-d_6) δ 160.48, 159.14, 158.02, 156.02, 155.37, 153.43, 151.87 (d, $J_{C\text{-}F}$ = 240.6 Hz), 145.28, 143.45, 142.40, 139.30 (d, $J_{C\text{-}F}$ = 10.8 Hz), 135.55 (d, $J_{C\text{-}F}$ = 9.3 Hz), 133.80, 133.54, 132.14 (2C), 128.51, 128.45 (2C), 128.10, 127.31, 127.13, 126.85, 121.57, 121.34, 116.94, 115.11, 107.90 (d, $J_{C\text{-}F}$ = 23.7 Hz), 99.45, 97.41, 86.60, 85.82, 57.96, 48.19, 21.71 (2C) ppm. LC-MS (ESI) m/z 637.00 [M + H]$^+$. HRMS (FAB) calculated for $C_{36}H_{29}FN_{10}O$ [M + H]$^+$: 637.2588, found: 637.2578.

N[2]-(4-((3-(4-(4-amino-1-ethyl-1H-pyrazolo[3,4-d]pyrimidin-3-yl)phenyl)prop-2-yn-1-yl)oxy)phenyl)-N[4]-(quinolin-3-yl)pyrimidine-2,4-diamine (3d)

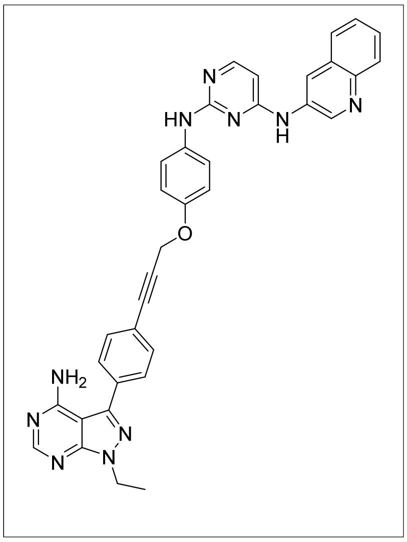

This compound was synthesized using the general procedure depicted in the method section (yield = 60.4%). ^{1}H NMR (400 MHz, DMSO-d_6) δ 9.83 (s, 1H), 9.17 (s, 1H), 8.95 (s, 1H), 8.93 (s, 1H), 8.25 (s, 1H), 8.08 (d, J = 5.7 Hz, 1H), 7.93 (d, J = 8.2 Hz, 1H), 7.81 (d, J = 6 Hz, 1H), 7.67-7.64 (m, 4H), 7.61-7.54 (m, 4H), 7.02 (d, J = 9 Hz, 2H), 6.31 (d, J = 5.7 Hz, 1H), 5.06 (s, 2H), 4.37 (q, J = 7.2 Hz, 2H), 1.40 (t, J = 7.2 Hz, 3H) ppm. ^{13}C NMR (100 MHz, DMSO-d_6) δ 160.44, 159.81, 158.12, 156.48, 155.68, 153.85, 152.33, 145.15, 143.31, 142.65, 134.50, 134.03, 133.33, 132.17 (3C), 128.54, 128.42 (3C), 128.19, 127.30, 126.98, 126.84, 121.54, 121.22, 115.05 (2C), 98.85, 97.29, 86.37, 86.05, 56.42, 41.44, 14.68 ppm. LC-MS (ESI) m/z 605.00 [M + H]$^+$. HRMS (FAB) calculated for $C_{35}H_{28}N_{10}O$ [M + H]$^+$: 605.2520, found: 605.2526.

N^2-(4-((3-(4-(4-amino-1-ethyl-1H-pyrazolo[3,4-d]pyrimidin-3-yl)phenyl)prop-2-yn-1-yl)oxy)-3-chlorophenyl)-N^4-(quinolin-3-yl)pyrimidine-2,4-diamine (3e)

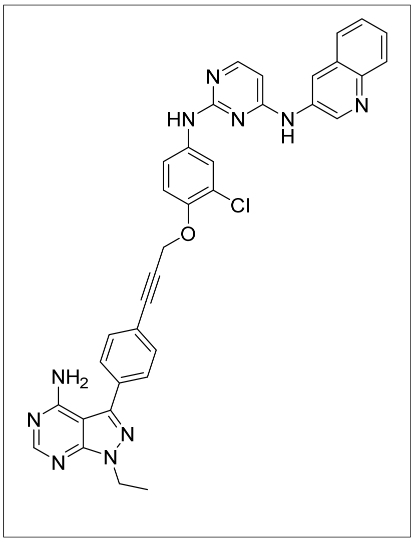

This compound was synthesized using the general procedure depicted in the method section (yield = 57.1%). ^{1}H NMR (400 MHz, DMSO-d_6) δ 9.89 (s, 1H), 9.34 (s, 1H), 8.96 (d, J = 2.2 Hz, 1H), 8.93 (s, 1H), 8.25 (s, 1H), 8.12 (d, J = 5.6 Hz, 1H), 7.96 - 7.92 (m, 2H), 7.85 (d, J = 7.3 Hz, 1H), 7.67 (d, J = 8.3 Hz, 2H) 7.63-7.52 (m, 5H), 7.27 (d, J = 9.0 Hz, 1H), 6.36 (d, J = 5.8 Hz, 1H), 5.15 (s, 2H), 4.37 (q, J = 7.2 Hz, 2H), 1.40 (t, J = 7.2 Hz, 3H) ppm. ^{13}C NMR (100 MHz, DMSO-d_6) δ 160.46, 159.33, 158.11, 156.34, 155.67, 153.85, 147.23, 145.26, 143.42, 142.62, 135.61, 133.92, 133.43, 132.21 (2C), 128.52, 128.44 (2C), 128.15, 127.43, 127.09, 126.82, 121.68, 121.43, 121.37, 120.83, 118.90, 115.46, 99.47, 97.29, 86.54, 85.79, 57.57, 41.44, 14.67 ppm. LC-MS (ESI) m/z 638.99 [M + H]$^+$. HRMS (FAB) calculated for $C_{35}H_{27}ClN_{10}O$ [M + H]$^+$: 639.2136, found: 639.2137.

N^2-(4-((3-(4-(4-amino-1-ethyl-1H-pyrazolo[3,4-d]pyrimidin-3-yl)phenyl)prop-2-yn-1-yl)oxy)-3-fluorophenyl)-N^4-(quinolin-3-yl)pyrimidine-2,4-diamine (3f)

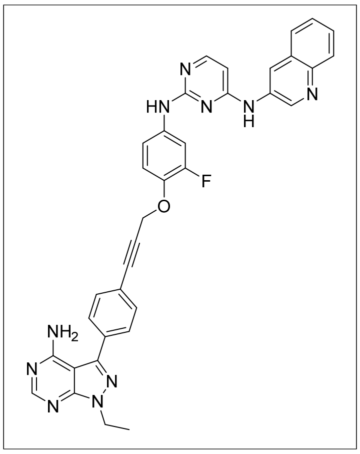

This compound was synthesized using the general procedure depicted in the method section (yield = 50%). ^{1}H NMR (400 MHz, DMSO-d_6) δ 9.89 (s, 1H), 9.38 (s, 1H), 8.96 (d, J = 2.2 Hz, 1H), 8.93 (brs, 1H), 8.25 (s, 1H), 8.12 (d, J = 5.7 Hz, 1H), 7.94 (d, J = 7.8 Hz, 1H), 7.86-7.82 (m, 2H), 7.67 (d, J = 8.2 Hz, 2H), 7.62-7.55 (m, 4H), 7.41 (d, J = 8.6 Hz, 1H), 7.26 (t, J = 9.3 Hz, 1H), 6.36 (d, J = 5.7 Hz, 1H), 5.12 (s, 2H), 4.37 (q, J = 7.2 Hz, 2H), 1.40 (t, J = 7.2 Hz, 3H) ppm. ^{13}C NMR (100 MHz, DMSO-d_6) δ 160.45, 159.32, 158.12, 156.39, 155.67, 153.85, 151.87 (d, J_{C-F} = 239.9 Hz), 145.28, 143.43, 142.63, 139.20 (d, J_{C-F} = 11 Hz), 135.69 (d, J_{C-F} = 9.8 Hz), 133.88, 133.41, 132.18 (2C), 128.54, 128.44 (2C), 128.14, 127.33, 127.12, 126.88, 121.46, 121.39, 116.92 (d, J_{C-F} = 2.1 Hz), 114.96, 107.76 (d, J_{C-F} = 22.5 Hz), 99.45, 97.29, 86.59, 85.86, 57.94, 41.44, 14.67 ppm. LC-MS (ESI) m/z 623.00 [M + H]$^+$. HRMS (FAB) calculated for $C_{35}H_{27}FN_{10}O$ [M + H]$^+$: 623.2432, found: 623.2435.

N^2-(3-((3-(4-(4-amino-1-isopropyl-1H-pyrazolo[3,4-d]pyrimidin-3-yl)phenyl)prop-2-yn-1-yl)oxy)phenyl)-N^4-(quinolin-3-yl)pyrimidine-2,4-diamine (4a)

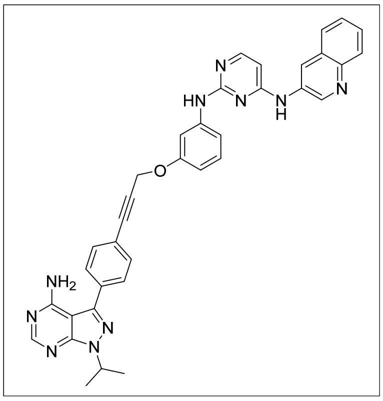

This compound was synthesized using the general procedure depicted in the method section (yield = 75.0%). ^{1}H NMR (400 MHz, DMSO-d_6) δ 9.89 (s, 1H), 9.37 (s, 1H), 9.01 (d, J = 1.9 Hz, 1H), 8.95 (d, J = 2.5 Hz, 1H), 8.24 (s, 1H), 8.14 (d, J = 5.7 Hz, 1H), 7.95-7.93 (m, 1H), 7.88-7.86 (m, 1H), 7.67 (d, J = 8.2 Hz, 2H), 7.62-7.56 (m, 5H), 7.36 (dd, J = 8.1 Hz, 1 Hz, 1H), 7.23 (t, J = 8.1 Hz, 1H), 6.72 (dd, J = 8 Hz, 2.2 Hz, 1H), 6.37 (d, J = 5.7 Hz, 1H), 5.06 (sep, 1H), 5.00 (s, 2H), 1.48 (d, J = 6.7 Hz, 6H) ppm. ^{13}C NMR (100 MHz, DMSO-d_6) δ 160.44, 159.46, 158.08, 157.73, 156.36, 155.45, 153.46, 145.12, 143.34, 142.42, 141.94, 133.94, 133.47, 132.16 (2C), 129.21, 128.50, 128.43 (2C), 128.20, 127.43, 127.04, 126.87, 121.49, 121.20, 112.63, 107.27, 106.43, 99.56, 97.42, 86.17, 86.04, 55.98, 48.18, 21.75 (2C) ppm. LC-MS (ESI) *m/z* 619.01 $[M + H]^+$. HRMS (FAB) calculated for $C_{36}H_{30}N_{10}O$ $[M + H]^+$: 619.2682, found: 619.2683.

N^2-(3-((3-(4-(4-amino-1-ethyl-1H-pyrazolo[3,4-d]pyrimidin-3-yl)phenyl)prop-2-yn-1-yl)oxy)phenyl)-N^4-(quinolin-3-yl)pyrimidine-2,4-diamine (4c)

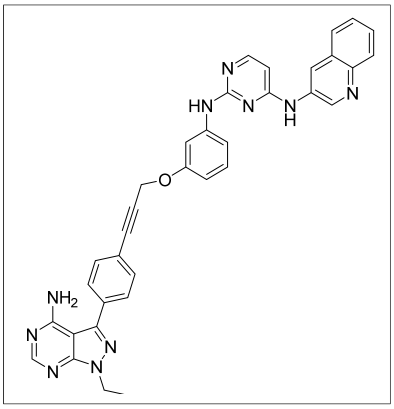

This compound was synthesized using the general procedure depicted in the method section (yield = 40.3%). ^{1}H NMR (400 MHz, DMSO-d_6) δ 9.90 (s, 1H), 9.37 (s, 1H), 9.01 (s, 1H), 8.95 (d, J = 2.4 Hz, 1H), 8.25 (s, 1H), 8.14 (d, J = 5.8 Hz, 1H), 7.95-7.86 (m, 2H), 7.66 (d, J = 8.2 Hz, 2H), 7.61-7.56 (m, 5H), 7.36 (d, J = 8 Hz, 1H), 7.23 (t, J = 8.1 Hz, 1H), 6.72 (dd, J = 8.1 Hz, 2 Hz, 1H), 6.37 (d, J = 5.7 Hz, 1H), 5.00 (s, 2H), 4.37 (q, J = 7.2 Hz, 2H), 1.40 (t, J = 7.2 Hz, 3H) ppm. ^{13}C NMR (100 MHz, DMSO-d_6) δ 160.44, 159.47, 158.13, 157.73, 156.39, 155.68, 153.86, 145.12, 143.34, 142.66, 141.95, 133.96, 133.33, 132.18 (2C), 129.22, 128.51, 128.41 (2C), 128.21, 127.44, 127.05, 126.88, 121.54, 121.19, 112.62, 107.25, 106.42, 99.57, 97.30, 86.20, 86.03, 55.98, 41.45, 14.69 ppm. LC-MS (ESI) *m/z* 605.00 $[M + H]^+$. HRMS (FAB) calculated for $C_{35}H_{28}N_{10}O$ $[M + H]^+$: 605.2526, found: 605.2518.

N^2-(3-((3-(4-(4-amino-1-ethyl-1H-pyrazolo[3,4-d]pyrimidin-3-yl)phenyl)prop-2-yn-1-yl)oxy)-4-methoxyphenyl)-N^4-(quinolin-3-yl)pyrimidine-2,4-diamine (4d)

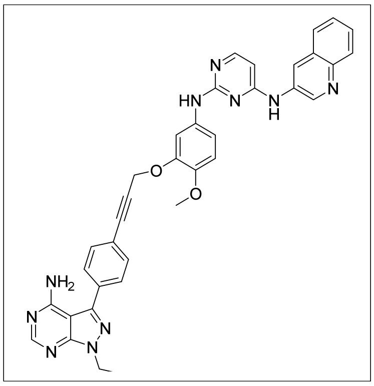

This compound was synthesized using the general procedure depicted in the method section (yield = 42.9%). ^{1}H NMR (600 MHz, DMSO-d_6) δ 9.82 (s, 1H), 9.17 (s, 1H), 8.95 (brs, 1H), 8.89 (s, 1H), 8.25 (s, 1H), 8.08 (d, J = 5.5 Hz, 1H), 7.92 (d, J = 8.2 Hz, 1H), 7.76 (brs, 1H), 7.63 (d, J = 8.3 Hz, 2H), 7.59-7.54 (m, 3H), 7.51 (d, J = 8.2 Hz, 2H), 7.30 (d, J = 7.8 Hz, 1H), 6.96 (d, J = 8.7 Hz, 1H), 6.30 (d, J = 6 Hz, 1H), 4.94 (s, 2H), 4.37 (q, J = 7.2 Hz, 2H), 3.81 (s, 3H), 1.40 (t, J = 7.4 Hz, 3H) ppm. ^{13}C NMR (150 MHz, DMSO-d_6) δ 160.40, 159.91, 158.11, 156.45, 155.65, 153.84, 146.50, 145.00, 144.72, 143.20, 142.63, 134.05, 134.03, 133.25, 132.10 (3C), 128.47, 128.30 (2C), 128.19, 127.31, 126.89, 126.75, 121.52, 121.05, 113.91, 112.57, 108.71, 98.87, 97.27, 86.14, 56.99, 55.92, 41.42, 14.64 ppm. LC-MS (ESI) m/z 635.00 [M + H]$^+$. HRMS (FAB) calculated for $C_{36}H_{30}N_{10}O_2$ [M + H]$^+$: 635.2631, found: 635.2625.

3-(p-tolyl)-1H-pyrazolo[3,4-d]pyrimidin-4-amine (1b)

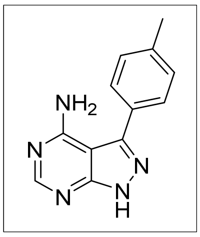

Compound **11b** (1.74 g, 8.8 mmol) was suspended in formamide (8 mL) and heated at 160 °C for 5h. The reaction mixture was cooled down to a room temperature, water was added, and the resulting precipitate was filtered to give a sand(brown) color solid (1.65 g, 7.3 mmol, yield = 83.4%). ^{1}H NMR (300 MHz, DMSO-d_6) δ 13.50 (brs, 1H), 8.20 (s, 1H), 7.55 (d, J = 8.1 Hz, 2H), 7.34 (d, J = 8.1 Hz, 2H), 2.38 (s, 3H) ppm. LC-MS (ESI) m/z 226.10 [M + H]$^+$. HRMS (FAB) calculated for $C_{12}H_{11}N_5$ [M + H]$^+$: 226.1093, found: 226.1087.

4-((4-(quinolin-3-ylamino)pyrimidin-2-yl)amino)phenol (23a)

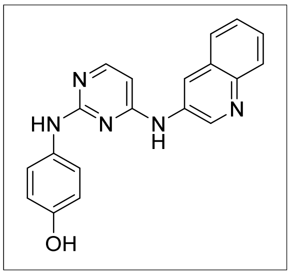

To a solution of **21** (500 mg, 1.9 mmol) in DMSO (1 mL) was added 4-aminophenol **22a** (233 mg, 2.1 mmol) and the mixture was stirred at 90 °C under N_2 atmosphere for 2 h. The mixture was cooled down to a room temperature, DCM was added, and the resulting precipitate was filtered to give a grey solid (580 mg, 1.8 mmol, 90.4% yield). ^{1}H NMR (300 MHz, DMSO-d_6) δ 9.77 (s, 1H), 9.12 (s, 1H), 8.96-8.94 (m, 2H), 8.90 (s, 1H), 8.05 (d, J = 5.7 Hz, 1H), 7.92 (d, J =

7.5 Hz, 1H), 7.76 (brs, 1H), 7.61-7.53 (m, 2H), 7.43 (d, J = 8.6 Hz, 2H), 6.72 (d, J = 8.4 Hz, 2H), 6.26 (d, J = 5.7 Hz, 1H) ppm. LC-MS (ESI) m/z 330.10 $[M + H]^+$. HRMS (FAB) calculated for $C_{19}H_{15}N_5O$ $[M + H]^+$: 330.1355, found: 330.1352.

N$_2$-(4-(prop-2-yn-1-yloxy)phenyl)-N$_4$-(quinolin-3-yl)pyrimidine-2,4-diamine (24a)

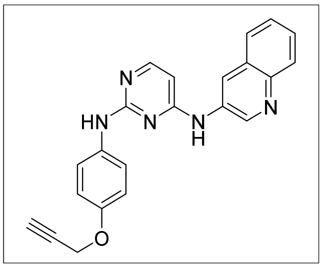

To a solution of **23a** (200 mg, 0.6 mmol) in anhydrous DMF (3 mL), were added cesium carbonate (396 mg, 1.2 mmol) and propargyl bromide (0.06 mL, 0.7 mmol). The reaction mixture was stirred at room temperature for 2 h. The distilled water was added and the precipitate was filtered and washed with water to give a grey solid (150 mg, 0.4 mmol, 67.2% yield). ^{1}H NMR (300 MHz, DMSO-d_6) δ 9.81 (s, 1H), 9.13 (s, 1H), 8.94 (brs, 1H), 8.91 (d, J = 2.4 Hz, 1H), 8.07 (d, J = 6 Hz, 1H), 7.94-7.91 (m, 1H), 7.80-7.77 (m, 1H), 7.62-7.53 (m, 4H), 6.93 (d, J = 9 Hz, 2H), 6.29 (d, J = 5.4 Hz, 1H), 4.76 (d, J = 2.4 Hz, 2H), 3.56 (t, J = 2.4 Hz, 1H) ppm. LC-MS (ESI) calculated for $C_{22}H_{17}N_5O$ $[M + H]^+$: 368.14, found: 368.20.

N$_2$-(3-chloro-4-(prop-2-yn-1-yloxy)phenyl)-N$_4$-(quinolin-3-yl)pyrimidine-2,4-diamine (24b)

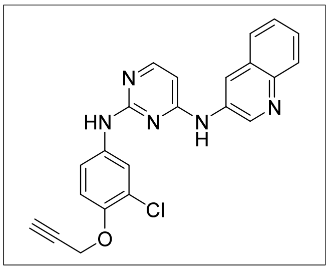

This compound was synthesized by using **23b**, yield = 59%. ^{1}H NMR (300 MHz, DMSO-d_6) δ 9.87 (s, 1H), 9.30 (s, 1H), 8.94 (d, J = 2.7 Hz, 1H), 8.92 (brs, 1H), 8.11 (d, J = 5.7 Hz, 1H), 7.94-7.91 (m, 2H), 7.82 (d, J = 8.7 Hz, 1H), 7.62-7.53 (m, 3H), 7.14 (d, J = 9 Hz, 1H), 6.34 (d, J = 6 Hz, 1H), 4.85 (d, J = 2.7 Hz, 2H), 3.61 (t, J = 2.3 Hz, 1H) ppm.

N$_2$-(3-fluoro-4-(prop-2-yn-1-yloxy)phenyl)-N$_4$-(quinolin-3-yl)pyrimidine-2,4-diamine (24c)

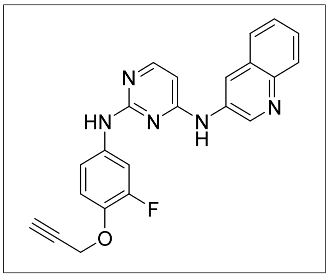

This compound was synthesized by using **23c**, yield = 61%. ^{1}H NMR (300 MHz, DMSO-d_6) δ 9.87 (s, 1H), 9.34 (s, 1H), 8.94 (d, J = 2.4 Hz, 1H), 8.92 (s, 1H), 8.11 (d, J = 5.7 Hz, 1H), 7.95-7.92 (m, 1H), 7.84-7.75 (m, 2H), 7.62-7.54 (m, 2H), 7.37 (d, J = 8.7 Hz, 1H), 7.14 (t, J = 9.3 Hz, 1H), 6.35 (d, J = 6 Hz, 1H), 4.82 (d, J = 2.4 Hz, 2H), 3.60 (t, J = 2.4 Hz, 1H) ppm.

N_2-(3-(prop-2-yn-1-yloxy)phenyl)-N_4-(quinolin-3-yl)pyrimidine-2,4-diamine (24d)

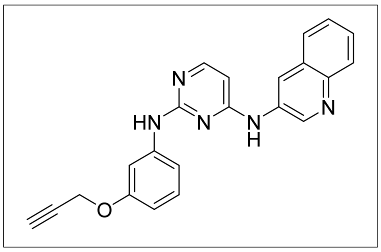

This compound was synthesized by using **23d**, yield = 62.8%. ^{1}H NMR (300 MHz, DMSO-*d_6*) δ 9.87 (s, 1H), 9.31 (s, 1H), 8.99 (d, *J* = 2.4 Hz, 1H), 8.94 (d, *J* = 2.7 Hz, 1H), 8.12 (d, *J* = 5.7 Hz, 1H), 7.94-7.83 (m, 2H), 7.62-7.54 (m, 2H), 7.46 (s, 1H), 7.37-7.34 (m, 1H), 7.19 (t, *J* = 8.3 Hz, 1H), 6.62 (dd, *J* = 8.1 Hz, 1.8 Hz, 1H), 6.36 (d, *J* = 5.7 Hz, 1H), 4.70 (d, *J* = 2.4 Hz, 2H), 3.54 (t, *J* = 2.4 Hz, 1H) ppm.

N_2-(4-methoxy-3-(prop-2-yn-1-yloxy)phenyl)-N_4-(quinolin-3-yl)pyrimidine-2,4-diamine (24e)

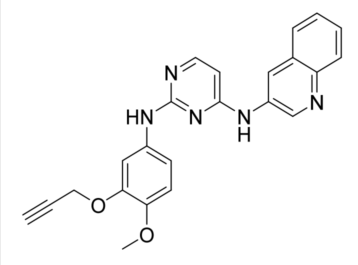

This compound was synthesized by using **23e**, yield = 60.3%. ^{1}H NMR (300 MHz, DMSO-*d_6*) δ 9.81 (s, 1H), 9.10 (s, 1H), 8.94 (s, 2H), 8.08 (d, *J* = 6 Hz, 1H), 7.93-7.90 (m, 1H), 7.78 (d, *J* = 6.6 Hz, 1H), 7.60-7.52 (m, 2H), 7.38 (s, 1H), 7.32 (dd, *J* = 8.7 Hz, 2.4 Hz, 1H), 6.91 (d, *J* = 8.7 Hz, 1H), 6.30 (d, *J* = 5.7 Hz, 1H), 4.64 (d, *J* = 2.1 Hz, 2H), 3.76 (s, 3H), 3.50 (t, *J* = 2.3 Hz, 1H) ppm.

III. ^{1}H and ^{13}C NMR spectra

^{1}H NMR of compound **3a** (500 MHz, DMSO-*d*$_6$)

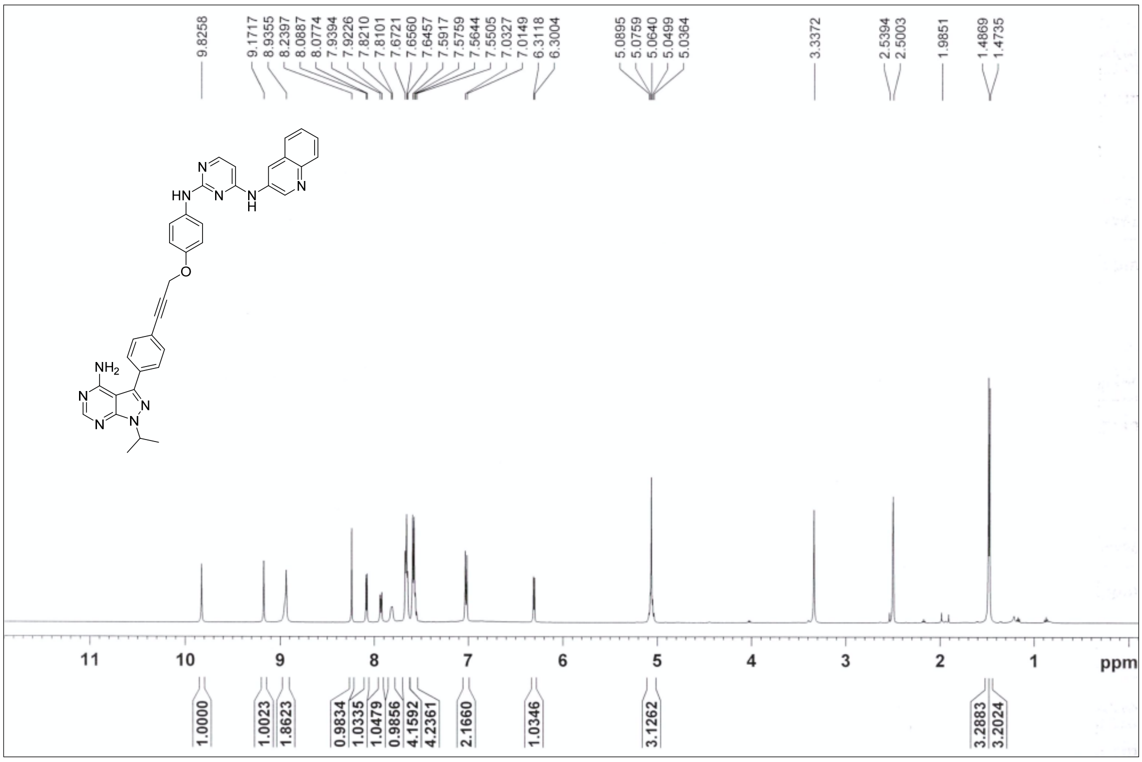

^{13}C NMR of compound **3a** (125 MHz, DMSO-*d*$_6$)

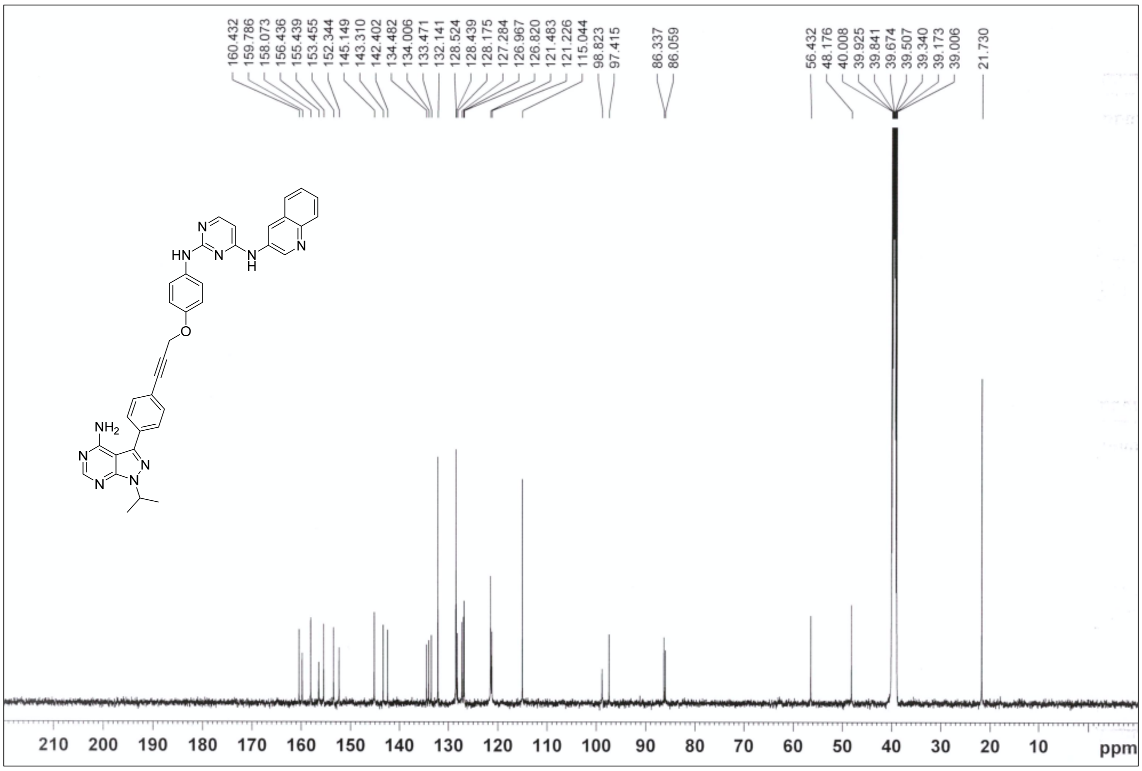

^{1}H NMR of compound **3b** (600 MHz, DMSO-d_6)

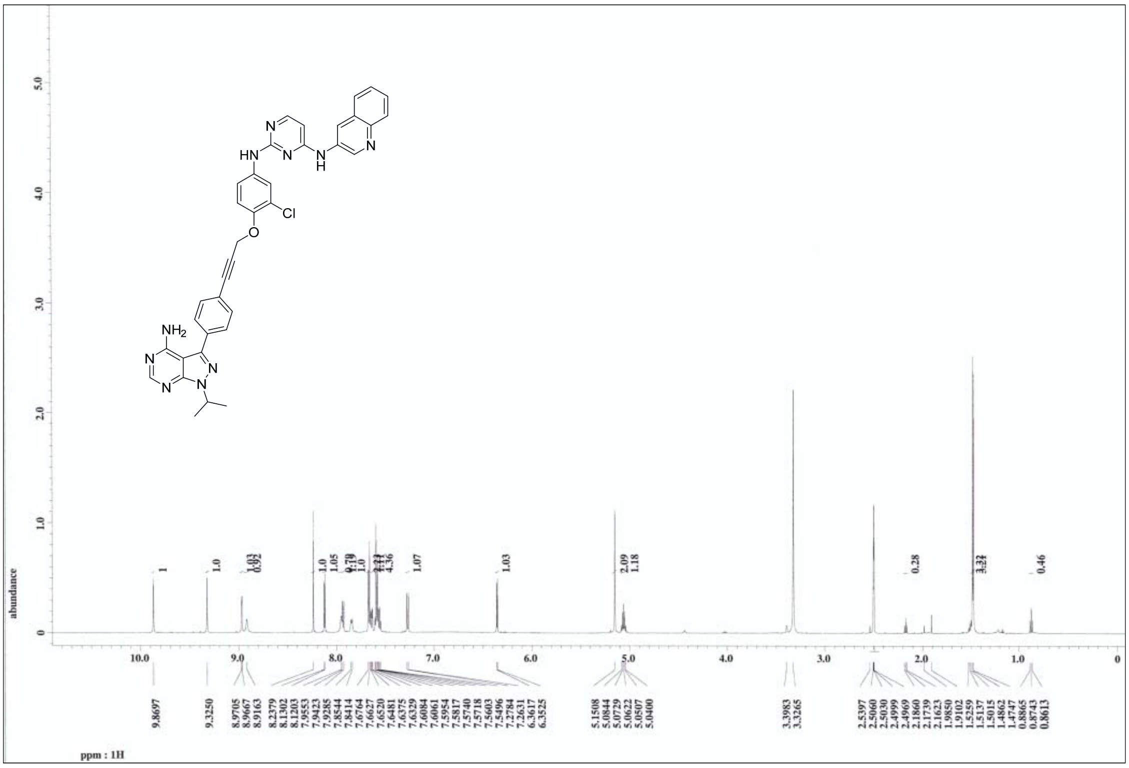

^{13}C NMR of compound **3b** (150 MHz, DMSO-d_6)

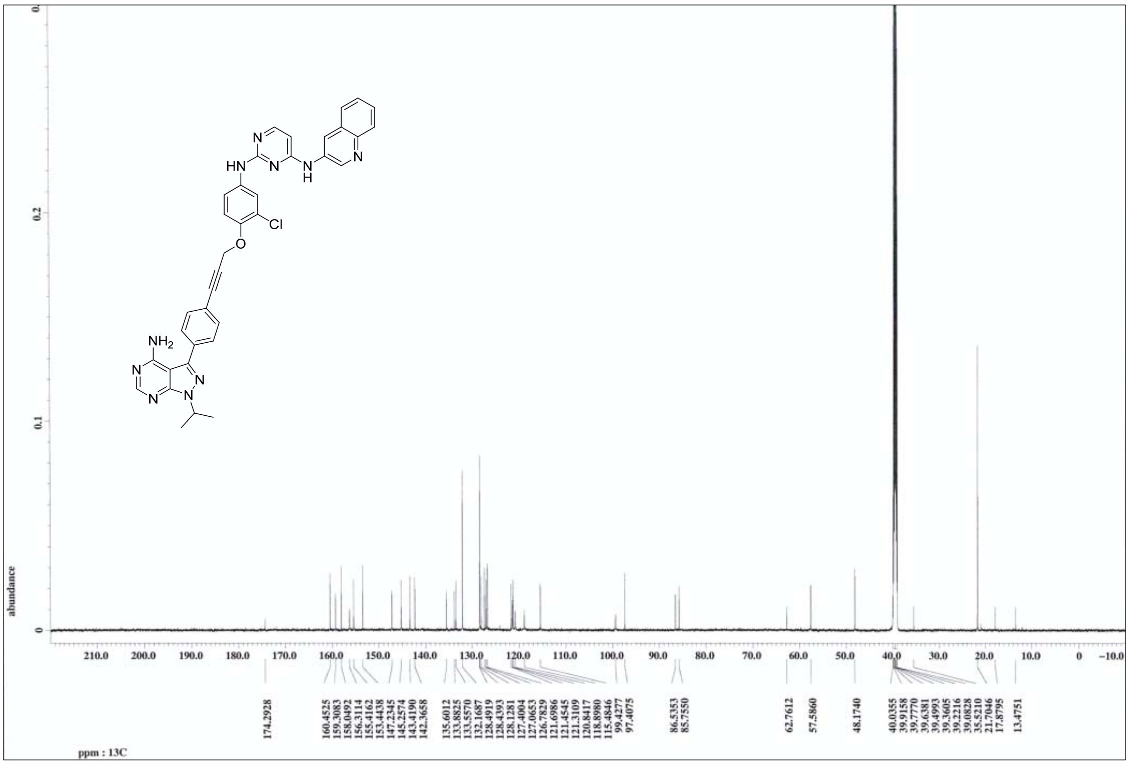

^{1}H NMR of compound **3c** (600 MHz, DMSO-d_6)

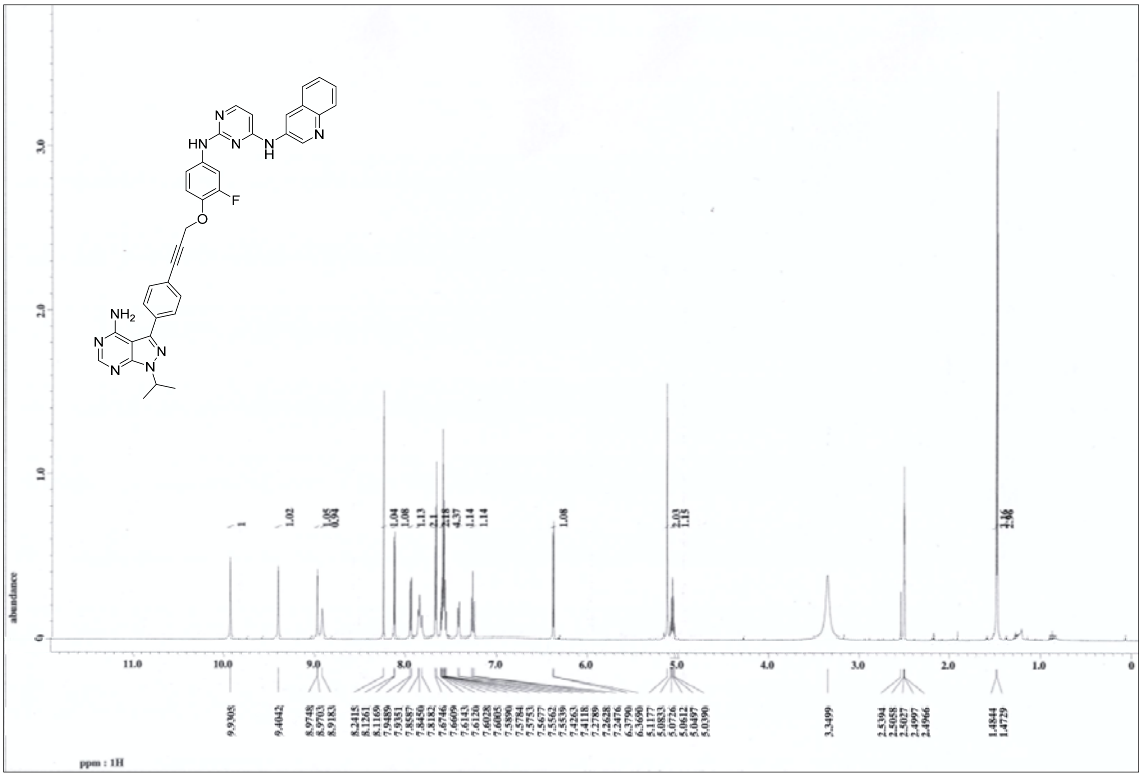

^{13}C NMR of compound **3c** (150 MHz, DMSO-d_6)

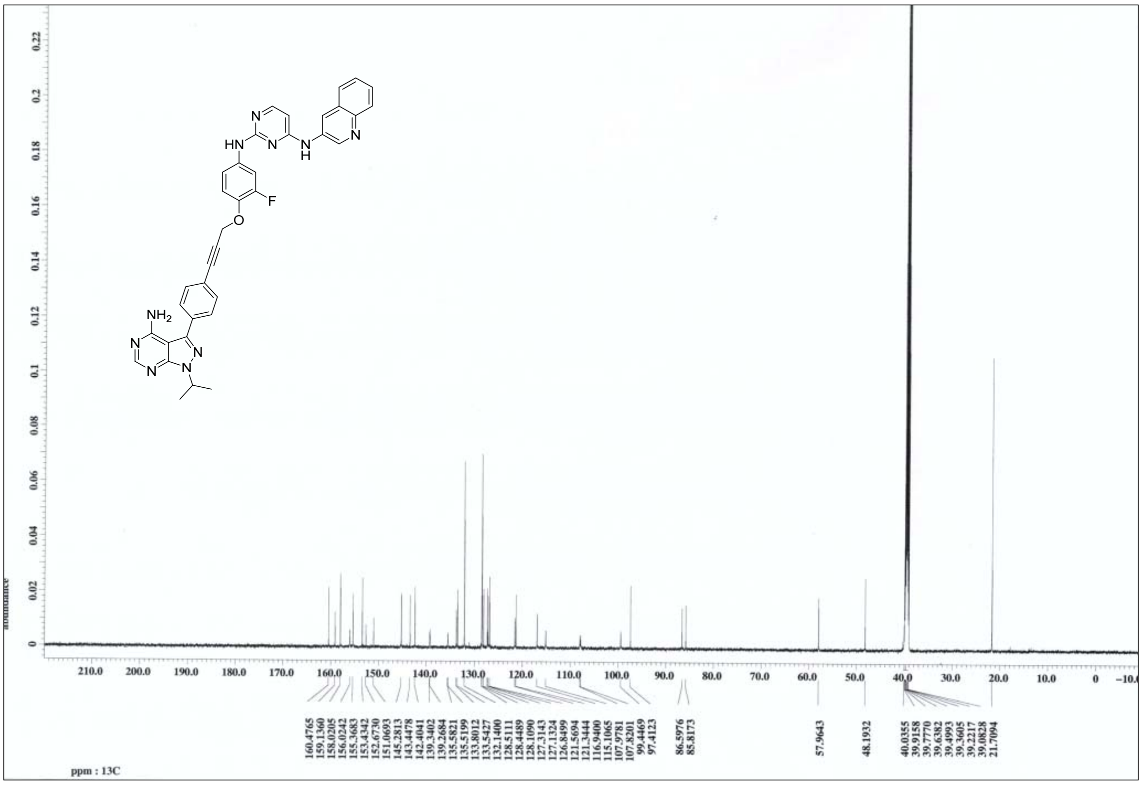

^{1}H NMR of compound **3d** (400 MHz, DMSO-d_6)

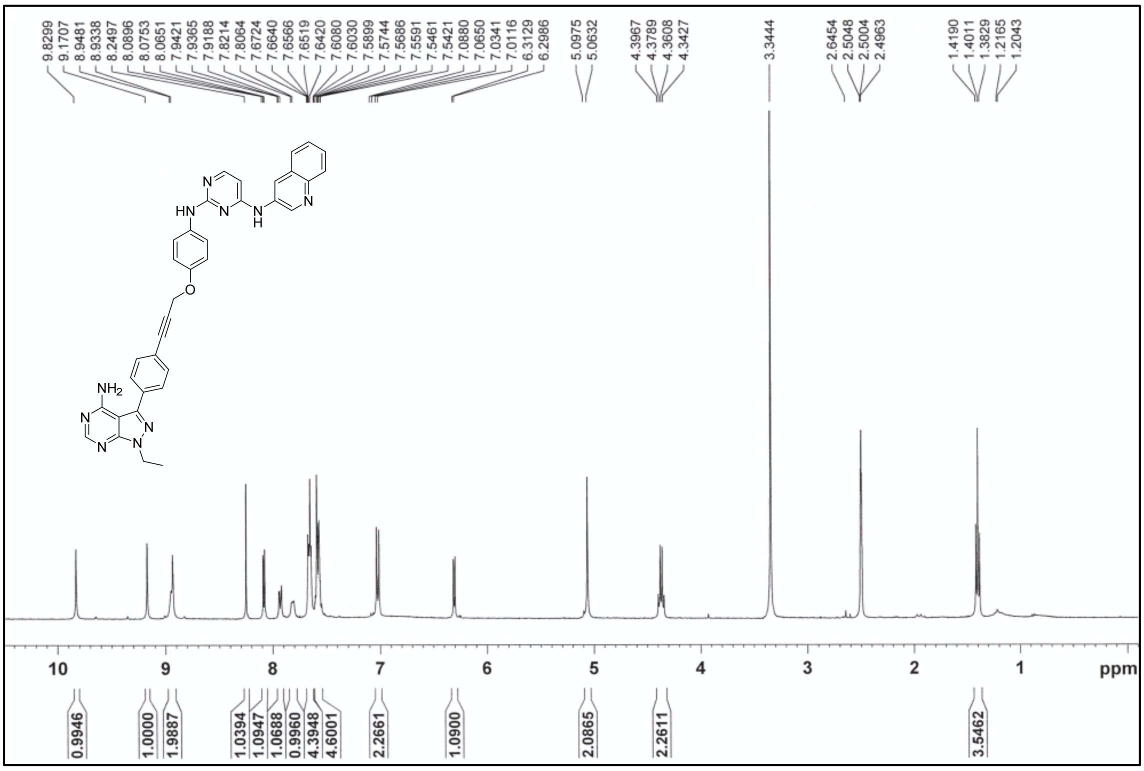

^{13}C NMR of compound **3d** (100 MHz, DMSO-d_6)

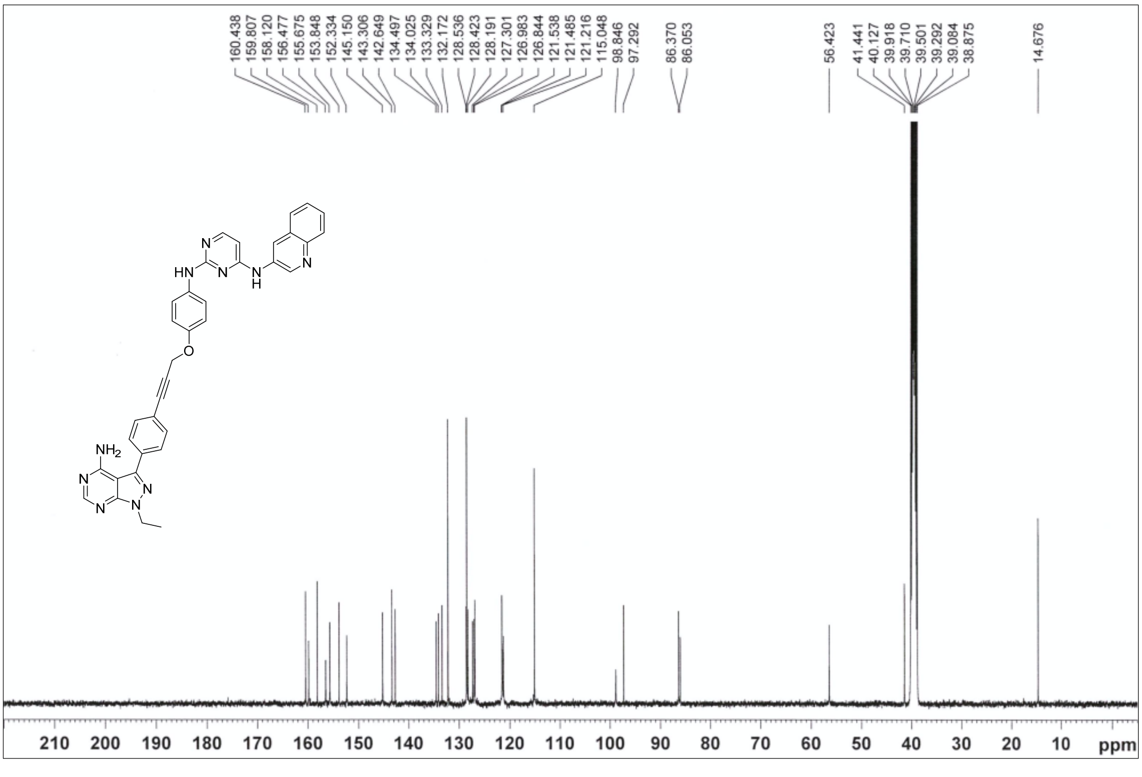

^{1}H NMR of compound **3e** (400 MHz, DMSO-d_6)

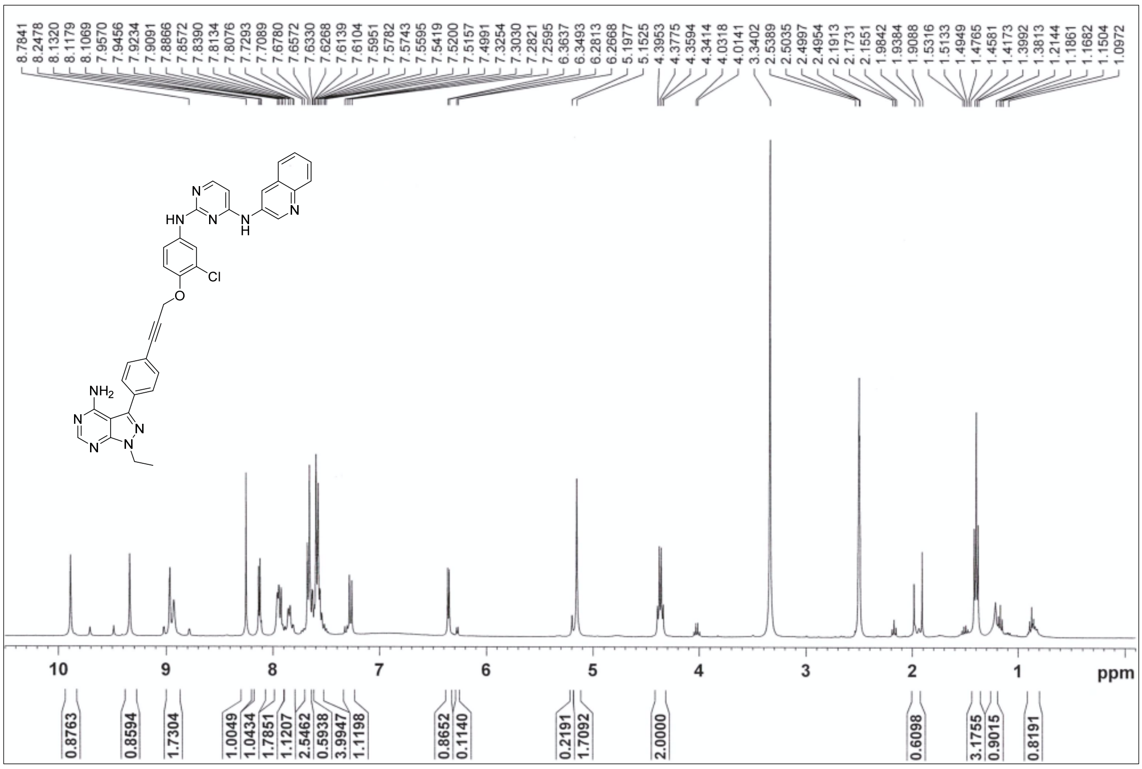

^{13}C NMR of compound **3e** (100 MHz, DMSO-d_6)

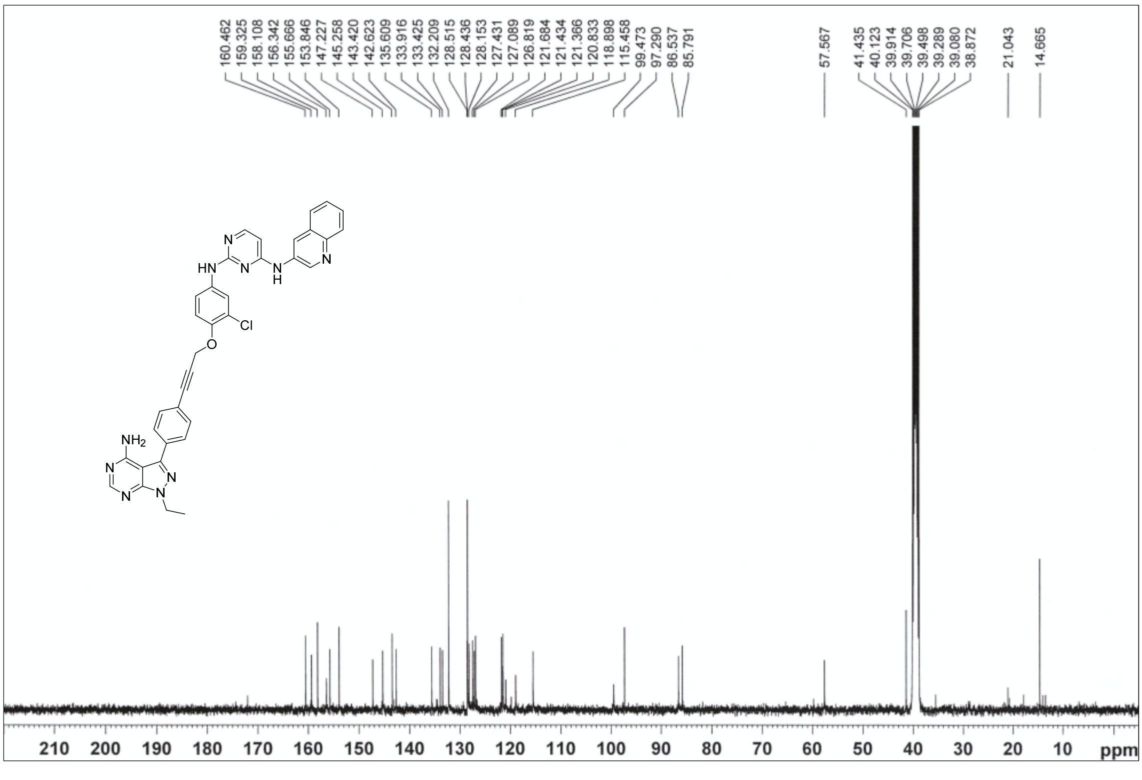

^{1}H NMR of compound **3f** (400 MHz, DMSO-d_6)

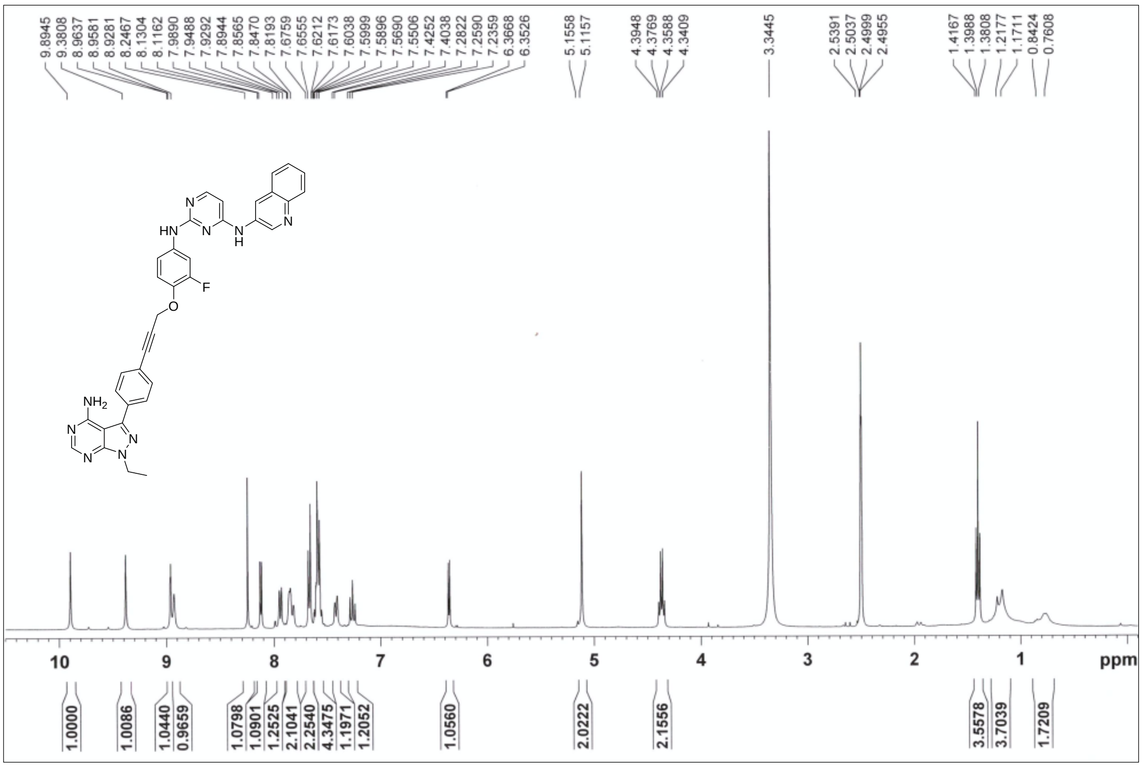

^{13}C NMR of compound **3f** (100 MHz, DMSO-d_6)

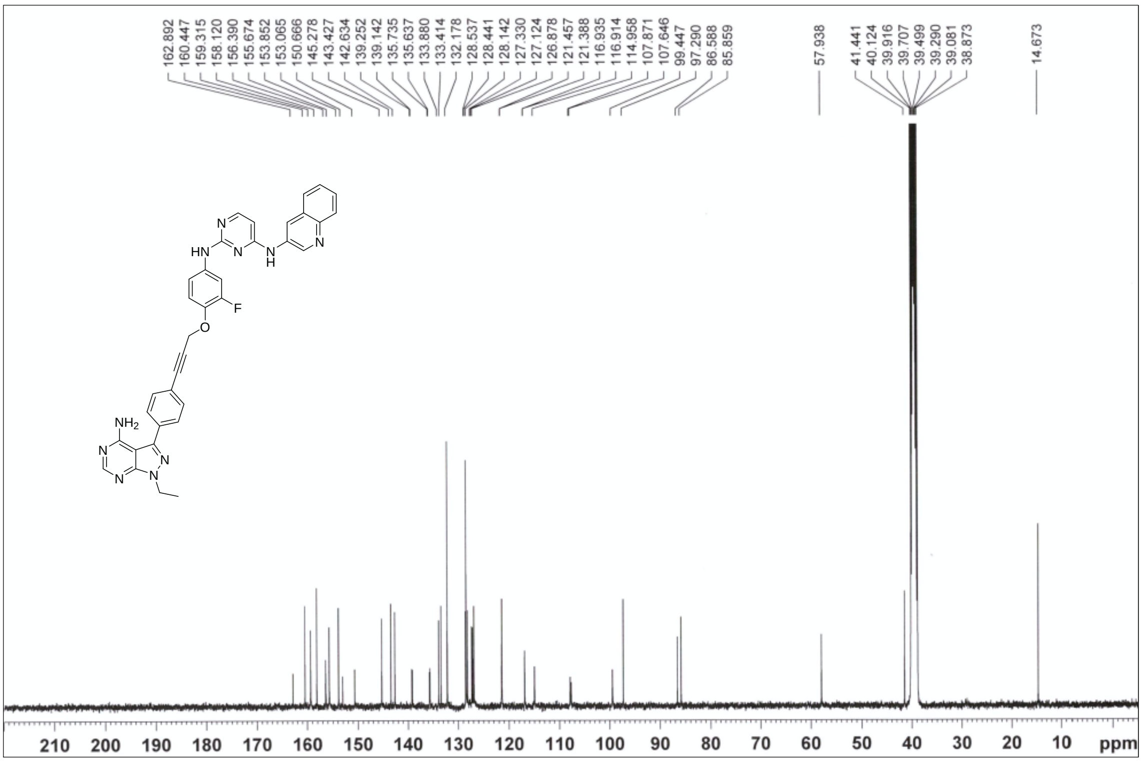

^{1}H NMR of compound **4a** (400 MHz, DMSO-d_6)

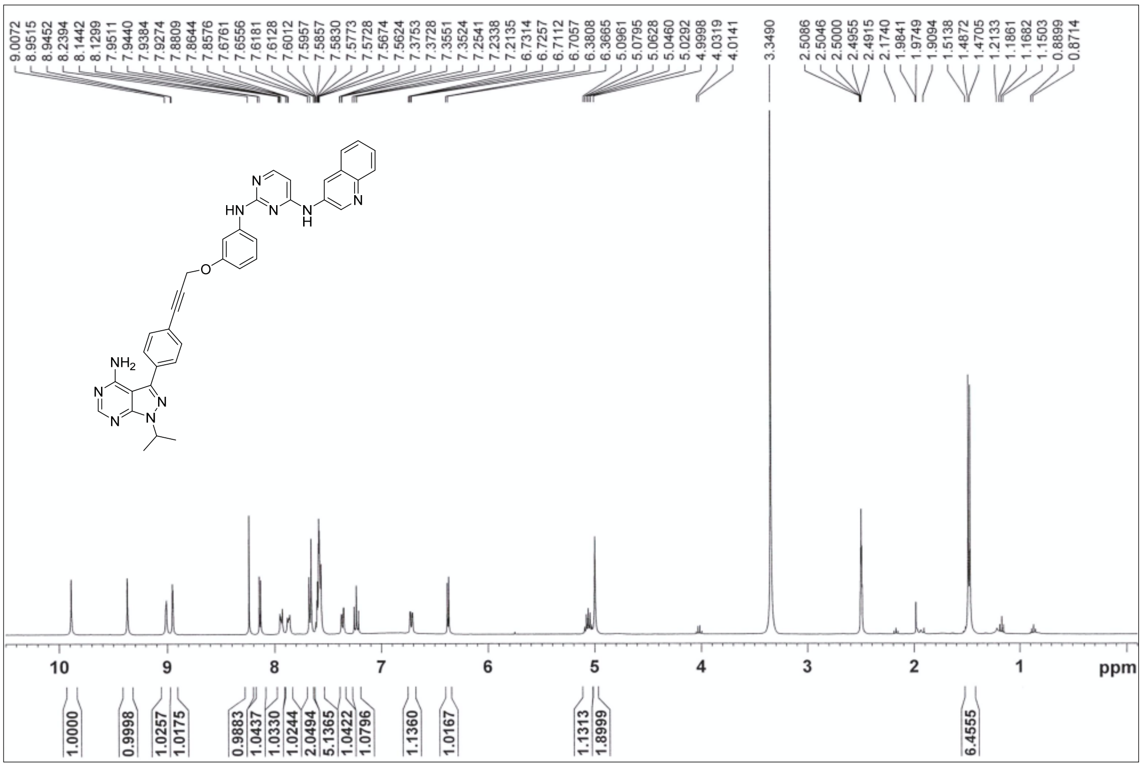

^{13}C NMR of compound **4a** (100 MHz, DMSO-d_6)

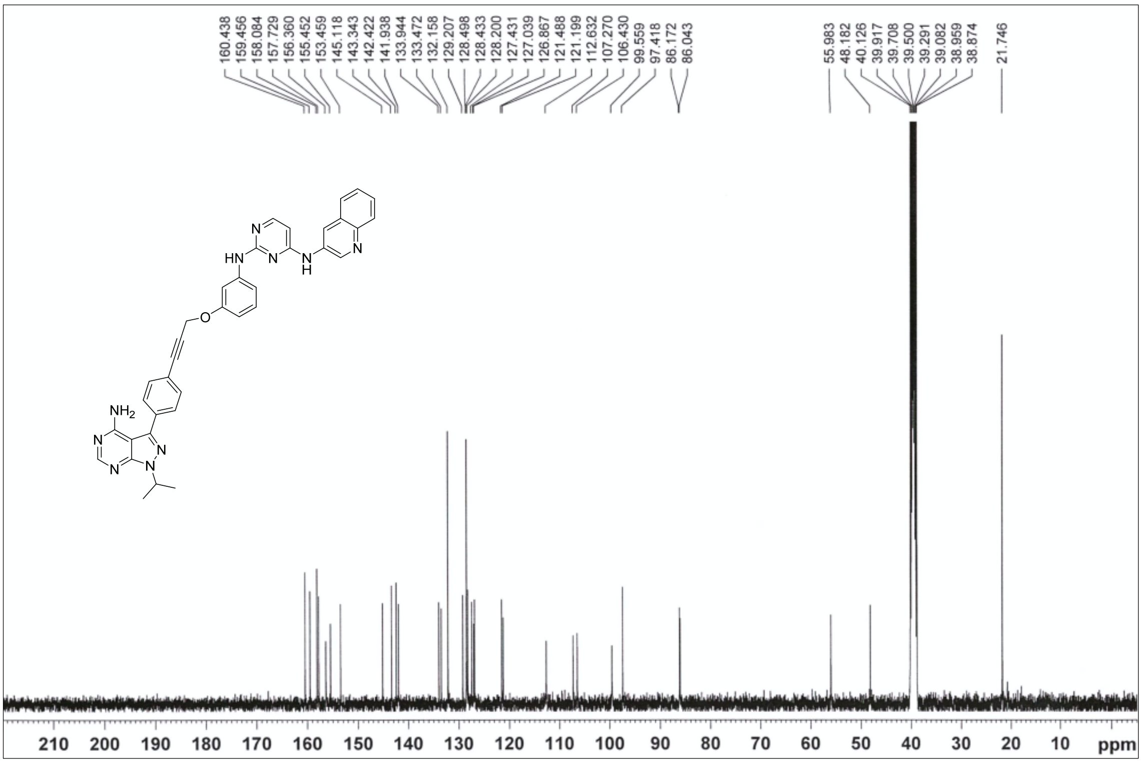

^{1}H NMR of compound **4b** (600 MHz, DMSO-d_6)

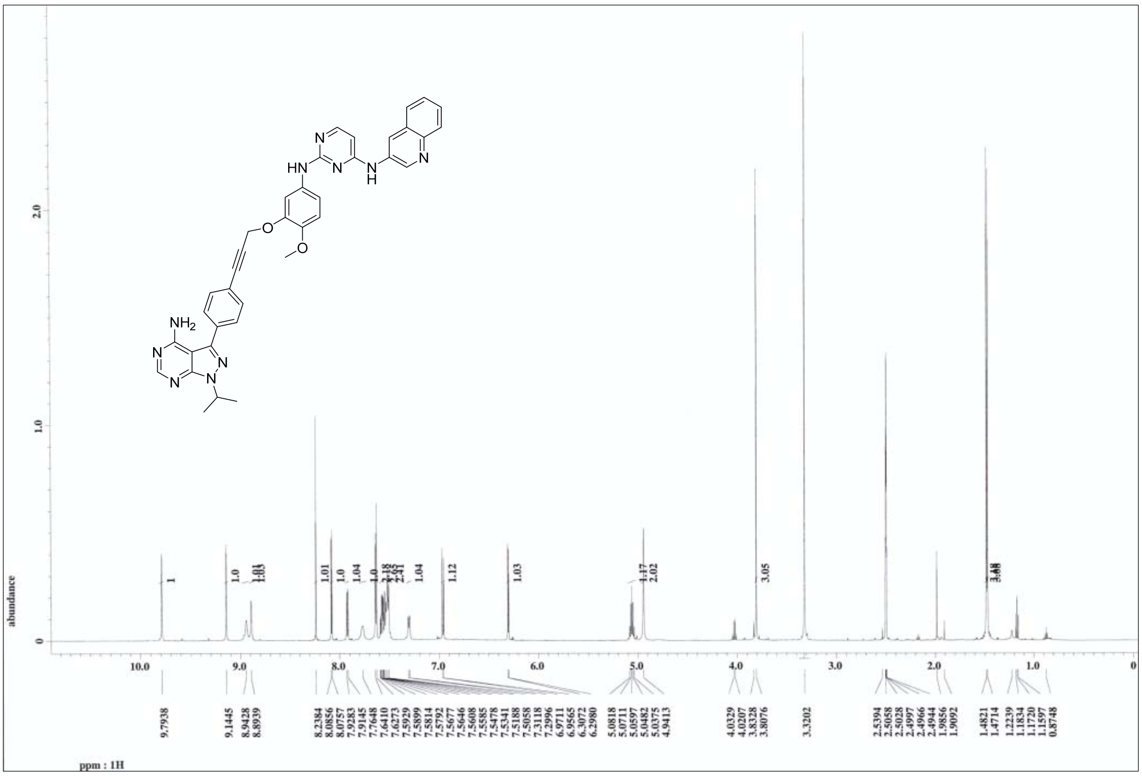

^{13}C NMR of compound **4b** (150 MHz, DMSO-d_6)

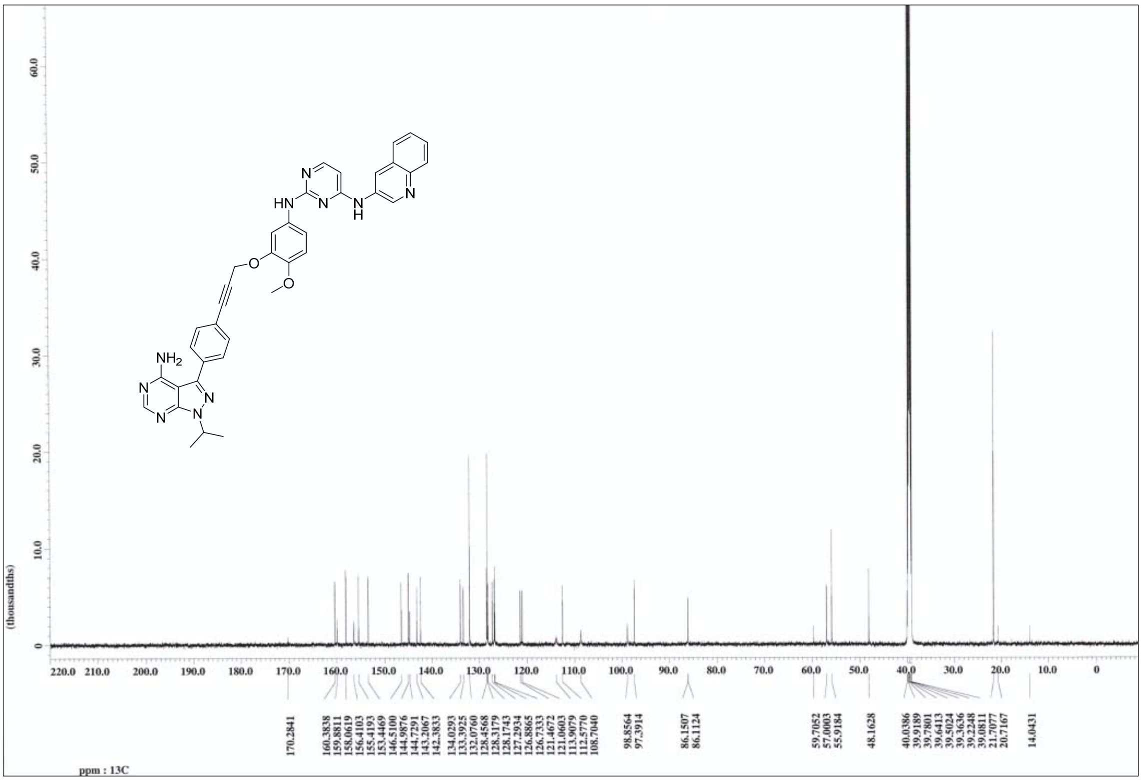

^{1}H NMR of compound **4c** (400 MHz, DMSO-d_6)

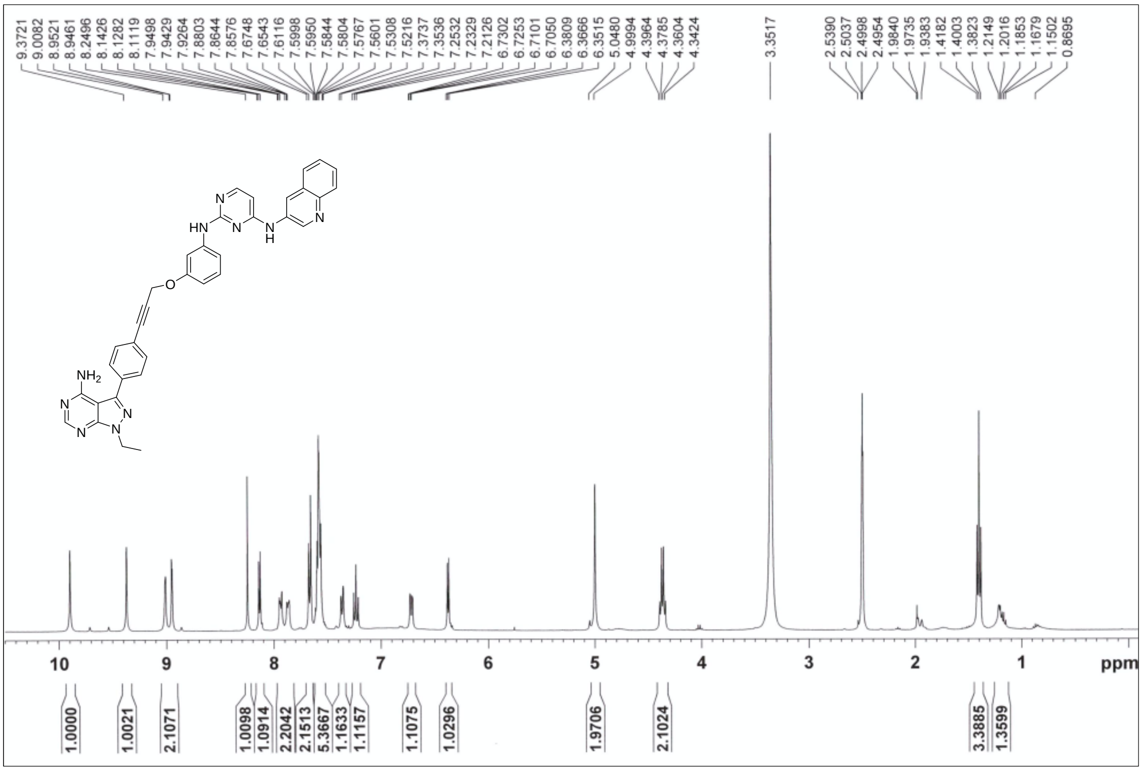

^{13}C NMR of compound **4c** (100 MHz, DMSO-d_6)

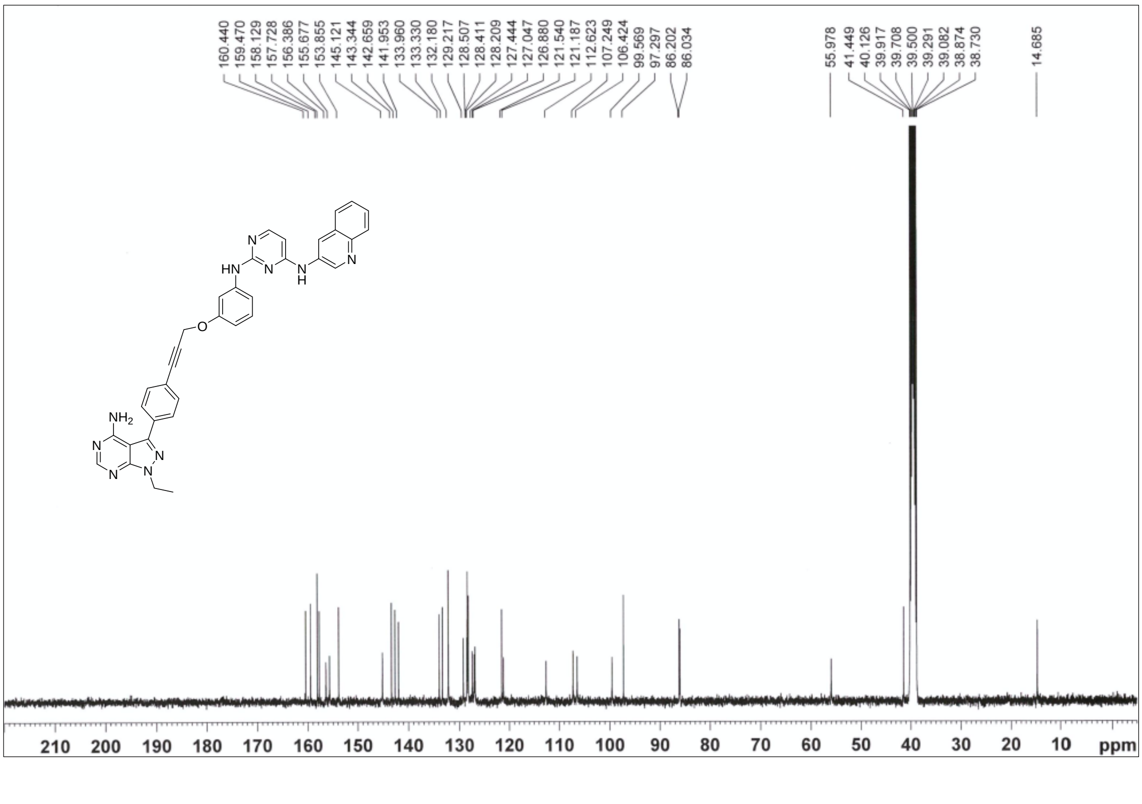

^{1}H NMR of compound **4d** (600 MHz, DMSO-d_6)

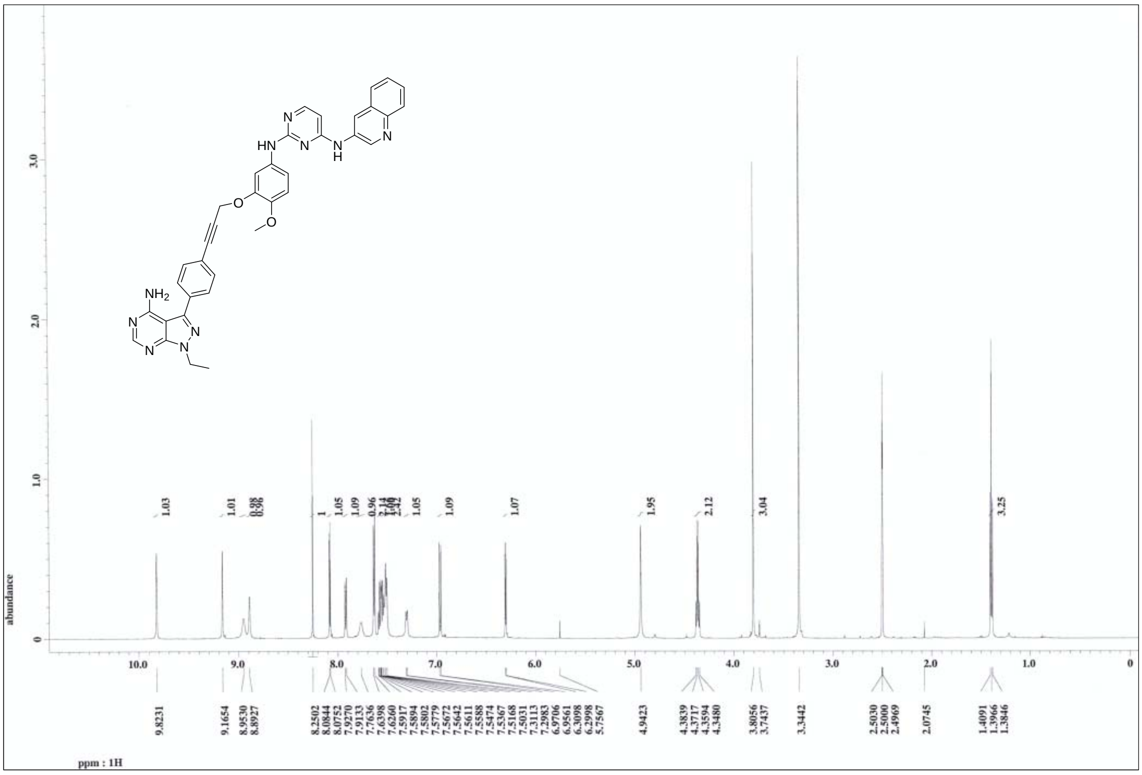

^{13}C NMR of compound **4d** (150 MHz, DMSO-d_6)

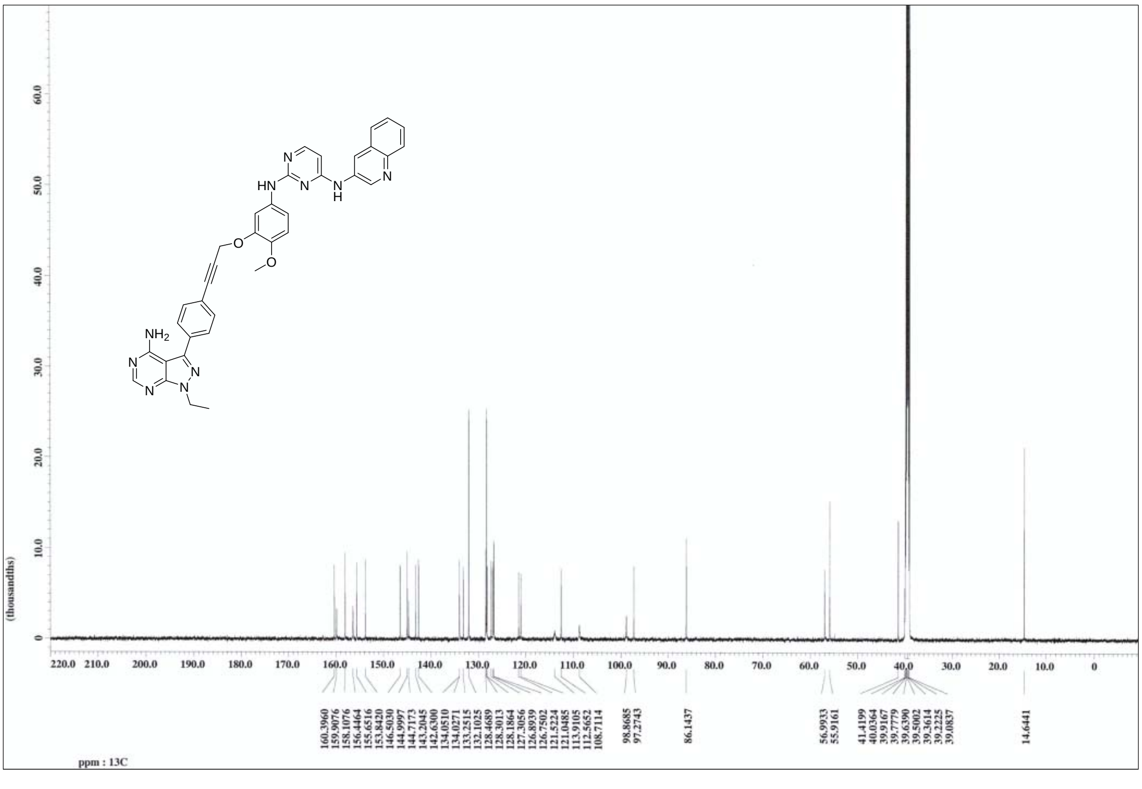

References

1. Kumar, A., et al., Synthesis and Evaluation of 3-Phenylpyrazolo[3,4-d]pyrimidine-Peptide Conjugates as Src Kinase Inhibitors. ChemMedChem, 2007. **2**(9): 1346-1360.
2. Buchanan, J.L., et al., Discovery of 2,4-bis-arylamino-1,3-pyrimidines as insulin-like growth factor-1 receptor (IGF-1R) inhibitors. Bioorg Med Chem Lett, 2011. **21**(8): 2394-9.
