# Supplementary material for: Development of a 4-aminopyrazolo[3,4-d]pyrimidine-based dual IGF1R/Src inhibitor as a novel anticancer agent with minimal toxicity
Source: Mol Cancer. 2018 Feb 19;17:50. doi: 10.1186/s12943-018-0802-4 (PMC5817804; doi:10.1186/s12943-018-0802-4)
Supplement: Supplementary file 2 — The IC50 values showing the inhibitory effect of selected compounds. (PDF 201 kb) [file 12943_2018_802_MOESM2_ESM.pdf]

Table S1. The IC_{50} values showing the inhibitory effect of selected compounds.

Compd	IC_{50} (μM)	
	A549	MCF-7
**3a**	3.7	4.6
**3c**	7.3	7.9
**4b**	3.6	4.0
**4d**	4.3	3.5
