# Supplementary material for: Development of a 4-aminopyrazolo[3,4-d]pyrimidine-based dual IGF1R/Src inhibitor as a novel anticancer agent with minimal toxicity
Source: Mol Cancer. 2018 Feb 19;17:50. doi: 10.1186/s12943-018-0802-4 (PMC5817804; doi:10.1186/s12943-018-0802-4)
Supplement: Supplementary file 3 — The IC50 values showing the inhibitory effect of LL28 on the viability of a panel of human lung cancer cells. (PDF 177 kb) [file 12943_2018_802_MOESM3_ESM.pdf]

Table S2. The IC_{50} values showing the inhibitory effects of LL28 on the viability of a panel of human lung cancer cells.

Cell line	IC_{50} (μM)
A549	1.16
H1299	1.61
H1944	0.97
H1993	0.57
H2170	0.76
H2122	1.36
H226B	0.62
H226Br	1.66
H460	0.70
H522	1.68
H596	0.74
HCC15	0.57
