# Supplementary material for: Development of a 4-aminopyrazolo[3,4-d]pyrimidine-based dual IGF1R/Src inhibitor as a novel anticancer agent with minimal toxicity
Source: Mol Cancer. 2018 Feb 19;17:50. doi: 10.1186/s12943-018-0802-4 (PMC5817804; doi:10.1186/s12943-018-0802-4)
Supplement: Supplementary file 4 — The IC50 values showing the inhibitory effect of LL28 on the anchorage-dependent colony -forming ability of a panel of human lung cancer cells. (PDF 176 kb) [file 12943_2018_802_MOESM4_ESM.pdf]

Table S3. The IC_{50} values showing the inhibitory effects of LL28 on the anchorage-dependent colony formation of a panel of human lung cancer cells.

Cell line	IC_{50} (μM)
A549	0.41
H1299	0.50
H1944	0.17
H2122	0.10
H226B	0.51
H226Br	0.50
H460	0.30
H2009	0.10
HCC15	0.17
