# Supplementary material for: Development of a 4-aminopyrazolo[3,4-d]pyrimidine-based dual IGF1R/Src inhibitor as a novel anticancer agent with minimal toxicity
Source: Mol Cancer. 2018 Feb 19;17:50. doi: 10.1186/s12943-018-0802-4 (PMC5817804; doi:10.1186/s12943-018-0802-4)
Supplement: Supplementary file 5 — Changes in the level of GPT, BUN, and creatinine by treatment with LL28 in mice. (PDF 109 kb) [file 12943_2018_802_MOESM5_ESM.pdf]

Table S4. Changes in the level of GPT, BUN, and creatinine by treatment with LL28 in mice.

Marker (unit)	Reference	LL28 (mg/kg)			
		0	20	40	80
GPT (U/l)	23-66	39 ± 2.12	37	62 ± 11.31	43 ± 1.41
BUN (mg/dl)	10-33	17.8 ± 4.45	20.7 ± 3.96	21.5 ± 1.34	27.8 ± 0.35
CREA (mg/dl)	0.156-0.231	0.1	0.2 ± 0.07	0.2 ± 0.07	0.2 ± 0.07
